# Comprehensive insights and In silico analysis into the emerging role of LincRNAs in lung diseases pathogenesis; a step toward ncRNA precision

**DOI:** 10.1007/s10142-025-01540-1

**Published:** 2025-02-06

**Authors:** Nadia M. Hamdy, Mohamed Bakr Zaki, Nourhan M. Abdelmaksoud, Shereen Saeid Elshaer, Mai A. Abd-Elmawla, Nehal I. Rizk, Doaa Fathi, Ahmed S. Doghish, Ahmed I. Abulsoud

**Affiliations:** 1 Biochemistry Department, Faculty of Pharmacy, Ain Shams University, Cairo, 11566 Abassia Egypt; 2 Department of Biochemistry, Faculty of Pharmacy, University of Sadat City, Sadat City, 32897 Menoufia Egypt; 3 Department of Biochemistry, Faculty of Pharmacy, Menoufia National University, Km Cairo-Alexandria Agricultural Road, Menoufia, Egypt; 4 Biochemistry Department, Faculty of Pharmacy, Heliopolis University, Cairo, 11785 Egypt; 5 Department of Biochemistry and Molecular Biology, Faculty of Pharmacy (Girls), Al Azhar University, Cairo, 11231 Nasr City Egypt; 6 Department of Biochemistry, Faculty of Pharmacy, Cairo University, Kasr Al-Ainy, Cairo, 11562 Egypt; 7 Department of Biochemistry, Faculty of Pharmacy and Drug Technology, Egyptian Chinese University, Cairo, 11786 Egypt; 8 Department of Biochemistry, Faculty of Pharmacy, Alexandria University, Alexandria, 21521 Egypt; 9 Department of Biochemistry, Faculty of Pharmacy, Badr University in Cairo (BUC), Cairo, 11829 Badr City Egypt; 10 Biochemistry and Molecular Biology Department, Faculty of Pharmacy (Boys), Al Azhar University, Cairo, 11231 Nasr City Egypt; 11 Faculty of Pharmacy, Integrative Health Centre, Heliopolis University, Cairo, 11785 Egypt

**Keywords:** NcRNA, LincRNAs, Lung carcinoma, LncRNAs, In silico, Precision medicine

## Abstract

Long non-coding RNAs (lncRNAs) have emerged as essential regulators of gene expression, significantly influencing various biological processes. Approximately half of all lncRNAs are classified as long intergenic non-coding RNAs (lincRNAs), which are situated among coding genes. Recent studies have documented the role of lincRNAs in the pathogenesis of lung diseases, including lung cancer, pulmonary fibrosis, and pulmonary arterial hypertension. These lincRNAs can modulate gene expression through various mechanisms, including epigenetic modifications, transcriptional regulation, and post-transcriptional regulation. By functioning as competing endogenous RNAs (ceRNAs), lincRNAs can affect the activity of microRNAs (miRNAs) and their corresponding target genes. This review delves into the intricate mechanisms by which lincRNAs contribute to the development and progression of various lung diseases. Furthermore, it discusses the potential of lincRNAs as therapeutic targets.

**Supplementary Information:**

The online version contains supplementary material available at 10.1007/s10142-025-01540-1.

## Introduction

The field of epigenetics, particularly the investigation of non-protein coding RNAs (ncRNAs), has lately experienced a significant surge in interest, especially following the joint awarding of the 2024 Nobel Prize in Physiology or Medicine for the discovery of microRNA (miRNA) (Lee et al. 1993; Wightman et al. 1993). While just a small fraction of mammals’ genome is translated into mRNAs that code for proteins, a staggering 80% of the human genome is involved in biochemical processes in some way, whether it’s RNA transcription, binding transcription factors, or changes to the arrangement of chromatin and histone modifications. ncRNAs are transcripts that originate from these noncoding regions; they cannot code for proteins (Wilusz et al. 2009; Abaza et al. 2023, Eldash et al. 2023, Hamdy et al. 2024c, Shaker et al. 2024).

Long noncoding RNA (lncRNA) is a noncoding transcript that is more than 200 nucleotides in length. Nearly half of all lncRNAs are long intergenic noncoding RNAs (lincRNAs), which have genes that are anywhere from a few nucleotides to over three Mb distant from the closest gene that codes for protein. They share features with mRNAs, including transcription by RNA polymerase II (RNAP2) and the ability to be spliced, capped, and polyadenylated (Hamdy et al. 2024c). However, lincRNAs are localized in the nucleus, expressed at a tenfold lower level than mRNAs, and primarily expressed in a manner that is particular to cell type, tissue, developmental stage, or disease state (Ransohoff et al. 2018).

The epigenetics of chromatin are controlled by lincRNAs. They affect transcription by forming R-loop structures at promoter regions and recruiting chromatin remodeling complexes. Functions include scaffolds for multiprotein complexes and RNAP2 recruitment and decoys. It may attach transcription factors to genomic targets. LincRNA loci can work in trans, allowing them to produce transcripts that can function at remote and unconnected genetic locations. Some lincRNA loci temporarily boost gene expression of neighboring genes. They control miRNAs, mRNA stability, translation, and posttranslational changes in the cytoplasm (Ransohoff et al. 2018, Kazimierczyk et al. 2020, Statello et al. 2021).

When lincRNA expression is out of whack, it can throw off the immunological and neurological systems in addition to disrupting cellular homeostasis, cell differentiation, and development. Cell proliferation, migration, invasion, apoptosis, epithelial-to-mesenchymal transition, cancer metastasis, and hereditary diseases may be influenced by lincRNA gene overexpression, lack, or mutation. Given their role in pathogenic responses, lincRNAs have frequently been found to regulate the expression of miRNAs and the genes that they target by acting as miRNA sponges. LincRNAs have a significant role in the development of a number of diseases. Potential therapeutic targets and useful indicators for predicting illness start, activity level or progression phase can be found in some lincRNAs (Quinn and Chang 2016, Kazimierczyk et al. 2020, Statello et al. 2021).

After 5′ capping, splicing, 3′ cleavage, and poly-adenylation, RNA polymerase II (Pol II) transcribes around 150,000 pre-mRNAs in the human genome. Although both mRNAs and lincRNAs undergo similar processing steps, lincRNAs are more prone to co-transcriptional cleavage and premature termination, whereas mRNAs exhibit more strong co-transcriptional splicing and polyadenylation. Some lincRNAs are spliced and polyadenylated like mRNAs, while others are cleaved and terminate transcription prematurely (Moore and Proudfoot 2009; Schlackow et al. 2017).

Every day brings more information regarding lincRNAs that may be involved in human diseases. There are pathophysiological functions for lincRNAs that are both protective and harmful. Their capacity to control fundamental cellular functions and cellular pathways for signaling is one manifestation of their dual nature (Plewka and Raczynska 2022).

## The different types of lung diseases

Lung diseases represent a significant global health challenge, affecting millions and encompassing a wide range of conditions that impair lung function. These diseases can be categorized into several types, including those affecting the airways, alveoli, interstitium, and chest wall. Common airway diseases include chronic obstructive pulmonary disease (COPD) and asthma, while lung cancer and pneumonia are notable conditions affecting the alveoli. Interstitial lung disease (ILD) and pneumoconiosis involve scarring of lung tissue, and various chest wall disorders can also impact breathing mechanics (Liao et al. 2019).

Lung cancer is a major global health issue and a leading cause of cancer-related deaths, primarily categorized into non-small cell lung cancer (NSCLC) and small cell lung cancer (SCLC). Adenocarcinoma, the most common subtype of lung cancer, accounts for about 40% of cases and is often found in non-smokers and younger individuals (Leiter et al. 2023).

In contrast, SCLC is a more aggressive form of lung cancer that grows rapidly and is almost exclusively associated with smoking (Saltos et al. 2020). It typically presents at an advanced stage, resulting in a poorer prognosis. Understanding the different subtypes of lung cancer and their potential for metastasis is essential for effective management and personalized treatment strategies.

Previously, unravelling the genetic component of various cancer types was researched heavily (Eissa, Swellam et al. 2003; El-Mesallamy et al. 2012, 2013; El Mesallamy et al. 2014; Youssef and Hamdy 2017; Ali et al. 2021), nowadays, epigenetics took over.

## LincRNAs as regulators in lung cancer and other pulmonary diseases

The dysregulation of ncRNA expression is associated with multiple illnesses, including lung disorders. LincRNAs share several regulatory mechanisms with mRNA, such as capping, polyadenylation, post-transcriptional modifications, and exonuclease degradation (Soni and Biswas 2021). These lincRNAs are dysregulated in lung diseases like lung cancer, acute lung injury, idiopathic pulmonary fibrosis, and PAH.

In recent years, lincRNA have become important regulators of chromatin modifications, alternative splicing, transcriptional and post-transcriptional regulation, mRNA stability, activity, and degradation. They also impact disease pathogenesis and cancer development (Ransohoff et al. 2018). Table 1 is demonstrating relationships between the pathophysiology of different lung diseases and lincRNA dysregulation. Table 2 depicts the interaction of lincRNAs with different molecular elements in various lung disease.

**Table 1:** List of human lincRNAs in different lung diseases, their dysfunctional type, locus, description of action or role

Lung disease	LincRNA	Alias	Dysfunction type	Description	Chr/Strand	Ref
Lung adenocarcinoma	LINC00511	Onco-lncRNA-12	Regulation	LAD cell growth was hampered by the up-regulated lncRNA LINC00511 being knocked down	17/-	(Wei and Zhang 2016)
	LINC00115	NCRNA00115	Regulation	Fundamental LAD therapeutic targets that act as ceRNAs to regulate miRNA-mRNA	1/-	(Li et al. 2016; Shao et al. 2021a; Xu and Nemati 2024)
	MALAT-1	HCN; LINC00047; NCRNA00047; NEAT2; PRO2853	Expression	Elevated in placenta previa increta/percreta and closely linked to in vitro invasion of trophoblast-like cells	11/ +	(Tseng et al. 2009)
	MEG3	FP504; GTL2; LINC00023; NCRNA00023; PRO0518; PRO2160; onco-lncRNA-83; prebp1	Regulation	Essential LAD therapeutic targets that act as ceRNAs to regulate miRNA-mRNA	14/ +	(Li et al. 2016)
	MIAT	C22orf35; GOMAFU; LINC00066; NCRNA00066; RNCR2			22/ +	
	MEG3	FP504; GTL2; LINC00023; NCRNA00023; PRO0518; PRO2160; onco-lncRNA-83; prebp1	Expression	Serves as a novel indicator of a poor response to cisplatin and may be a target for LAD treatment	14/ +	(Liu et al. 2015)
	RP11-325I22.2	LINC01511	Regulation	Participates in the process by which lung cancer is caused by the loss of EGFR exon 19	3/-	(Wang et al. 2014)
Lung cancer	BCYRN1	BC200; BC200a; LINC00004; NCRNA00004	Expression	Expressed in carcinomas of the breast, cervix, esophagus, lung, ovary, parotid, and tongue, but not in the corresponding normal tissues	2/ +	(Chen et al. 1997)
	H19	ASM; ASM1; BWS; D11S813E; LINC00008; NCRNA00008; WT2	Expression	H19 downregulation dramatically reduces anchorage-independent proliferation and clonogenicity in breast and lung cancer cells. In primary breast and lung carcinomas, there is a substantial correlation between MYC and H19 expression. Controls the metastatic process of NSCLC	11/-	(Barsyte-Lovejoy et al. 2006; Wang et al. 2016a, b)
			Regulation	Controls of the colon, endometrium, prostate, esophagus, cervix, and breast imprinting. Management of imprinting with miR-675		(Qi and Du 2013; Jiang and Bikle 2014)
			Expression	Estimates the risk of lung cancer and the response to platinum-based chemotherapy		(Gong et al. 2016a)
	MALAT-1	HCN; LINC00047; NCRNA00047; NEAT2; PRO2853	Interaction	Enhances lung cancer cell motility by controlling the transcription and post-transcriptional expression of genes linked to motility	11/ +	(Tano et al. 2010)
			Expression	Overexpressed in HCC, cervical cancer, and lung cancer		(Li et al. 2014)
			Expression	Predictor for the emergence of lung cancer metastases		(Hrdlickova et al. 2014)
			Regulation	Controls alternative splicing by sequestering SR splicing factors		(Qi and Du 2013)
			Regulation	Important modulator of lung cancer cells’ metastatic phenotype		(Gutschner et al. 2013a)
			Regulation	Controls invasion and metastasis in neuroblastoma, osteosarcoma, colon, prostate, liver, and cervix cancers		(Jiang and Bikle 2014)
			NA	Silencing MALAT-1 could be a useful treatment strategy for tumors		(Ren et al. 2016)
			Expression	Predicts the risk of lung cancer and the response to platinum-based chemotherapy		(Gong et al. 2016a)
			Expression	Assesses lung cancer and shows how the host reacts to it		(Guo et al. 2015a, b, c)
			Expression	The transcription factor Sp1 upregulates A549 lung cancer cells; Sp1 may be a target for cancer treatment		(Li et al. 2015a, b)
			Regulation	Performs a crucial part in lung cancer metastasis and triggers lung cancer cell migration		(Tee et al. 2014)
			Regulation	Important modulator of lung cancer cells’ metastatic phenotype		(Gutschner et al. 2013a)
	MEG3	FP504; GTL2; LINC00023; NCRNA00023; PRO0518; PRO2160; onco-lncRNA-83; prebp1	Expression	Expression is lost	14/ +	(Zhang et al. 2003)
	CCAT2	LINC00873; NCCP1	Expression	Predicts the risk of lung cancer and the response to platinum-based chemotherapy	8/ +	(Gong et al. 2016a)
	FENDRR	FOXF1-AS1; TCONS_00024240; lincFOXF1; onco-lncRNA-21	Expression	Used to identify a new treatment target or to diagnose Xuanwei lung cancer (XWLC)	16/-	(Li et al. 2015a, b)
	BANCR	LINC00586	Regulation	Reduction of BANCR expression increased the ability of cells to survive radiation	9/-	(Chen et al. 2015)
				Enhances lung cancer migration and growth through MAPK pathways		(Jiang et al. 2015)
Lung cancer brain metastasis	MALAT-1	HCN; LINC00047; NCRNA00047; NEAT2; PRO2853	Regulation	May be a promising prognostic factor and therapeutic target to treat lung cancer brain metastases in the future since it induces EMT, which promotes lung cancer brain metastases	11/ +	(Shen et al. 2015)
Lung squamous cell carcinoma	LINC01133	N/A	Expression	Elevated in LSCC and serves as a predictor for survival	1/ +	(Zhang et al. 2015)
	PVT-1	LINC00079; MYC; NCRNA00079; onco-lncRNA-100	Regulation	LSCC cell proliferation was suppressed by PVT-1 knockdown	8/ +	(Wei and Zhang 2016)
			Regulation	Its regulators and targets could be very important to LSCC’s development		(Wu, Ruan et al. 2016a, b)
Non-small cell lung cancer	BANCR	LINC00586	Expression	BANCR downregulation affects EMT, which encourages metastasis and is associated with poor prognosis for NSCLC	9/-	(Sun et al. 2014)
	CCAT2	LINC00873; NCCP1	Regulation	Fosters NSCLC invasion	8/ +	(Qiu et al. 2014)
	MALAT-1	HCN; LINC00047; NCRNA00047; NEAT2; PRO2853	Expression	Expression is strongly associated with a worse outcome and increased metastasis	11/ +	(Wapinski and Chang 2011)
			Expression	Elevated expression in metastatic NSCLC and predicts survival and metastasis in early-stage NSCLC		(Ji et al. 2003)
			Expression	Acts as a predictor of early-stage NSCLC and tumor prognosis		(Rotblat et al. 2011)
			Expression	Causes migration and tumor growth and is indicative of a bad prognosis in NSCLC		(Schmidt et al. 2011a)
			Expression	Reported in NSCLC by its overexpression		(Harries 2012)
			Regulation	Controls the migration, invasiveness, and viability of tumor cells		(Shuai et al. 2016)
			Expression	Plays a key role in the bone metastases of NSCLC and dramatically increases migration, invasion, and tumorigenesis in vivo		(Liu et al. 2016)
			N/A	Appropriate for the early diagnosis of NSCLC		(Peng et al. 2016)
			Locus	Shown to be carcinogenic and linked to tumor invasion in NSCLC that is controlled by DNA methylation		(Guo et al. 2015a, b, c)
			Expression	It is a complementary factor in a panel that enhances the overall diagnostic performance		(Weber et al. 2013)
			Regulation	Bcl-2’s predictive effect was discovered to be dependent on MALAT-1 expression		(Schmidt et al. 2014)
			Expression	NSCLC cellular proliferation, migration, and invasion are regulated by MALAT-1 expression through TDP 43 regulation		(Guo et al. 2015a, b, c)
	TUG-1	LINC00080; NCRNA00080; TI-227H	Regulation	By epigenetically controlling HOXB7 expression, p53-regulated TUG-1 influences cell proliferation in human NSCLC	22/ +	(Zhang et al. 2014)
			Expression	Downregulated in NSCLC and has the ability to control *CELF1* when it binds to PRC2		(Lin et al. 2016)
	FENDRR	FOXF1-AS1; TCONS_00024240; lincFOXF1; onco-lncRNA-21	Regulation	FOXF1-AS1 loss controls NSCLC metastasis, stemness, and EMT	16/-	(Miao et al. 2016)
	XIST	DXS1089; DXS399E; LINC00001; NCRNA00001; SXI1; swd66	Locus	Suppresses KLF2 expression epigenetically, acting as an oncogene in NSCLC	X/-	(Fang et al. 2016)
	NEAT1	LINC00084; NCRNA00084; TncRNA; VINC	Regulation	Stimulates the growth of NSCLC by controlling the miR-377-3p-E2F3 pathway	11/ +	(Sun et al. 2016)
				Functions as a competitive endogenous lncRNA in NSCLC to sponge hsa-mir 98-5p and increase EGCG-induced CTR1		(Jiang et al. 2016)
	TUSC7	LINC00902; LSAMP-AS1; LSAMP-AS3; LSAMPAS3; NCRNA00295	Expression	Serves in the prognosis of NSCLC, and TUSC7 dysregulation may be a significant factor in the development of NSCLC	3/ +	(Wang et al. 2016a, b)
	PVT-1	LINC00079; MYC; NCRNA00079; onco-lncRNA-100	Regulation	PVT-1/EZH2/LATS2 interactions may be a novel target for the diagnosis and treatment of lung cancer	8/ +	(Wan et al. 2016a)
	H19	ASM; ASM1; BWS; D11S813E; LINC00008; NCRNA00008; WT2	Regulation	MYC -activated H19 enhances the cell cycle progression of NSCLC via downregulating miR-107	11/-	(Cui et al. 2015)
			Expression	In NSCLC, MYC -regulated H19 impacts cell proliferation and is indicative of a bad prognosis		(Zhang et al. 2016)
			Regulation	Acts as a diagnostic tool, an oncogenic regulator of NSCLC, and a target for novel treatments in NSCLC patients		(Zhang et al. 2016)
	UCA1	CUDR; LINC00178; NCRNA00178; UCAT1; onco-lncRNA-36	Locus	One of the mechanisms by which this oncogene in NSCLC upregulates *ERBB4* is by sponging miR-193a-3p	19/ +	(Nie et al. 2016)
			Regulation	Activates the AKT/mTOR pathway in EGFR-mutant NSCLC, causing non-T790M acquired resistance to EGFR-TKIs		(Cheng et al. 2015)
			Regulation	Contributes to the development of lung cancer and serves in clinical diagnosis		(Wang et al. 2015)
	PVT-1	LINC00079; MYC; NCRNA00079; onco-lncRNA-100	Expression	Help in NSCLC diagnosis and prognosis	8/ +	(Cui et al. 2016)
			Regulation	In NSCLC, elevated expression encourages carcinogenesis		(Yang et al. 2014)
	MEG3	FP504; GTL2; LINC00023; NCRNA00023; PRO0518; PRO2160; onco-lncRNA-83; prebp1	Regulation	MEG3 and miR-3163 work together to limit *SKP2* mRNA translation in NSCLC cells, which stops the proliferation of NSCLC cells	14/ +	(Su et al. 2016)
				Contributes to NSCLC’s development of cisplatin resistance		(Xia et al. 2015)
	NEAT1	LINC00084; NCRNA00084; TncRNA; VINC	Expression	Circulating ANRIL, NEAT, and SPRY4-IT1 are predictors for the early detection of NSCLC	11/ +	(Hu et al. 2016)
			Regulation	Linked to NSCLC development and oncogenesis, and it implies use in molecular targeted therapy		(Pan et al. 2015)
	RP11-397D12.4	LINC01627	Expression	RP11-397D12.4, AC007403.1, and ERICH1-AS1 are possible predictors for NSCLC tumorigenesis in the future	9/-	(Tang et al. 2015)
	AC007403.1	LINC01628			2/-	
	XIST	DXS1089; DXS399E; LINC00001; NCRNA00001; SXI1; swd66	Expression	Elevated serum HIF1A-AS1 and XIST could predict NSCLC	X/-	(Tantai et al. 2015)
	BCYRN1	BC200; BC200a; LINC00004; NCRNA00004	Regulation	A marker induced by MYC that controls NSCLC cell metastasis	2/ +	(Hu and Lu 2015)
	SNHG1	LINC00057; NCRNA00057; U22HG; UHG; lncRNA16	Regulation	Stimulates the growth of cells in NSCLC	11/-	(You et al. 2014)
	HMlincRNA717	N/A	Expression	A possible therapeutic target, and independent prognostic factor for NSCLC patients	N/A/ N/A	(Xie et al. 2014)
	BANCR	LINC00586	Regulation	A predictor for poor NSCLC prognosis and an EMT regulator during NSCLC metastasis	9/-	(Sun et al. 2014)
	CCAT2	LINC00873; NCCP1	Regulation	Helps NSCLC invade and is a biomarker for lymph node metastasis	8/ +	(Qiu et al. 2014)
	LINC-RoR	N/A	Regulation	Higher levels of LINC-ROR are associated with advanced TNM stage, positive distant metastasis, and lymph node metastasis		(Qu et al. 2017)
Pneumoconiosis	H19	ASM; ASM1; BWS; D11S813E; LINC00008; NCRNA00008; WT2	Mutation	The preventive effects of coal workers’ pneumoconiosis in a Chinese population are influenced by polymorphisms in H19	11/-	(Wu et al. 2016a)
	lncRNA-ATB	N/A	Expression	Associated with the risk of pneumoconiosis in coal miners and could be useful for its identification	N/A/ N/A	(Cui et al. 2016)
Pulmonary arterial hypertension	MALAT-1	HCN; LINC00047; NCRNA00047; NEAT2; PRO2853	Regulation	Chinese people are more susceptible to PAH due to MALAT-1 polymorphism	11/ +	(Zhuo et al. 2017)
Small cell lung cancer	CCAT2	LINC00873; NCCP1	Regulation	Controls growth and metastasis in SCLC and forecasts a poor prognosis	8/ +	(Chen et al. 2016)
	LINC00173		Expression	High levels of LINC00173 correlate with advanced disease stages and poor prognosis in SCLC patients and are linked to shorter survival rates	12/	(Zeng et al. 2020)

Retrieved from lncRNAdisease database https://www.cuilab.cn/lncrnadisease Accessed September 26th, 2023 and LncRNAWiki 2.0 Community curation of lincRNAs for integrating human lincRNAs with multi-omics annotations https://ngdc.cncb.ac.cn/lncbook/omics/expression Accessed September 27th, 2023.

[LincRNA: Long intergenic non-coding RNA; LAD: Lung adenocarcinoma; ceRNAs: Competing endogenous RNA; miRNA: MicroRNA; mRNA: Messenger RNA; NCRNA: Non-coding RNA; MALAT-1: Metastasis associated lung adenocarcinoma transcript 1; NEAT2: Nuclear-enriched abundant transcript 2; MEG3: Maternally expressed gene 3; MIAT: Myocardial infarction associated transcript; EGFR: Epidermal growth factor receptor; BCYRN1: Brain Cytoplasmic RNA 1; NSCLC: Non-small cell lung cancer; HCC: Hepatocellular carcinoma; SR: Serine/arginine-rich; NA: Not available; Sp1: Specificity protein 1; CCAT2: Colon cancer-associated transcript 2; FENDRR: Fetal-lethal non-coding developmental regulatory RNA; XWLC: Xuanwei lung cancer; BANCR: BRAF activated non-coding RNAs; MAPK: Mitogen-activated protein kinase; EMT: Epithelial-mesenchymal transition; PVT-1: Plasmacytoma Variant Translocation 1; LSCC: Lung squamous cell carcinoma; Bcl-2: B-cell lymphoma 2; TDB 43: TAR-DNA-binding Protein 43; TUG-1: Taurine-upregulated gene 1; HOXB7: Homeobox B7; CELF1: CUGBP and Elav-like family member 1; FOXF1-AS1: Forkhead Box F1 Antisense 1; XIST: X inactive specific transcript; KLF2: Krüppel-like Factor 2; NEAT1: Nuclear enriched abundant transcript 1; EGCG: Epigallocatechin-3-gallate; CTR1: Copper transporter 1; TUSC7: Tumor suppressor candidate 7; EZH2: Enhancer of zeste homolog 2; LATS2: Large tumor suppressor kinase 2; UCA1: Urothelial cancer associated 1; *ERBB4*: Erb-b2 receptor tyrosine kinase 4; TKIs: Tyrosine kinase inhibitors; *SKP2*: S-phase kinase-associated protein 2; ANRIL: Antisense Noncoding RNA in the INK4 Locus; SPRY4-IT1: Sprouty RTK signaling antagonist 4-intronic transcript 1; ERICH1-AS1: Glutamate-Rich 1; HIF1A-AS1: Hypoxia inducible factor 1 subunit alpha-antisense 1; SNHG1: Small nucleolar RNA host gene 1; lncRNA-ATB: lncRNA-activated by TGF-β; SCLC: Small cell lung cancer.]

**Table 2:** LINC interactions partner, level, and type of interactions in lung cancer

LincRNA	Interaction partner(s)	Level of interaction	Type of interaction	Reference
LINC00037 or DGCR5	REST	DNA-TF	Regulatory	(Johnson et al. 2009)
LINC00008 or H19	IGF2	RNA-RNA	Co-expression	(Clark and Mattick 2011)
	CTCF1, CTCF2 & CTCF3	RNA-RNA	Co-expression	(Pidsley et al. 2012)
	miR-675	RNA-RNA	Co-expression	(Ponting et al. 2009)
	GLI1 & SHH	DNA-TF	Regulatory	(Qureshi et al. 2010)
	p53	DNA-TF	Regulatory	(Qureshi et al. 2010)
	MYC	DNA-TF	Regulatory	(Qureshi et al. 2010)
	E2F1	RNA–Protein	Regulatory	(Qureshi et al. 2010)
LINC00064, or HAR1	RELN	DNA-TF	Regulatory	(Pollard et al. 2006)
	REST	DNA-TF	Regulatory	(Johnson et al. 2010)
LINC00078: HULC	miR-372	RNA-DNA	Regulatory	(Wang et al. 2010)
		RNA-RNA	Binding/Regulatory	(Wang et al. 2010)
LINC-RoR	Nanog, Sox2, Oct4	DNA-TF	Regulatory	(Loewer et al. 2010)
LINC00312, or ERR-10	ERA	RNA-DNA	Regulatory	(Ju et al. 2009)
LINC00570, or ncRNA-a5	ROCK2	RNA-RNA	Co-expression	(Ørom et al. 2010)
LINC00047 or MALAT-1	CREB	DNA-TF	Regulatory	(Koshimizu et al. 2010)
	IBP160, RNPS1 & SRm160	RNA–Protein	Regulatory	(Miyagawa et al. 2012)
	Pc2	DNA–Protein	Regulatory	(Yang et al. 2011)
	SR, SRSF1 proteins	RNA–Protein	Binding	(Tripathi et al. 2010)
LINC00023, or MEG3	cAMP & CRE	DNA-TF	Regulatory	(Zhao et al. 2006)
	GDF15	RNA-DNA	Regulatory/binding	(Zhou et al. 2007)
	p53	RNA-DNA	Regulatory/co-expression	(Zhou et al. 2007; Zhang et al. 2010)
LINC00066, MIAT	IRES-GFP	DNA-DNA	Regulatory	(Rapicavoli et al. 2010)
LINC00084, NEAT1	DBHS	RNA–Protein	Binding	(Bond and Fox 2009)
	P54NRB/NONO & PSF/SFPQ, PSPC1	RNA–Protein	Binding	(Bond and Fox 2009)
	p54nrb & PSF	RNA–Protein	Binding	(Nakagawa et al. 2011)
PVT-1	MYC	DNA-TF & RNA-DNA	Regulatory	(Carramusa et al. 2007)
	YY1	RNA-DNA	Regulatory	(Meyer et al. 2011)
	p53	DNA-TF	Regulatory	(Barsotti et al. 2012)
LINC00096, TP53TG1	*TP53*	DNA-TF	Regulatory	(Takei et al. 1998)
TUG-1	p53	DNA-TF	Regulatory	(Khalil et al. 2009)
	PRC2	RNA–Protein	Binding	(Khalil et al. 2009)
	Pc2	DNA–Protein	Regulatory	(Yang et al. 2011)
LINC00001, or XIST	PRC2	RNA–Protein	Binding	(Zhao et al. 2008)
	TAP/NXF1	RNA–Protein	Binding	(Cohen and Panning 2007)

### ANRIL

Antisense noncoding RNA in the INK4 Locus (ANRIL) was found to be elevated in NSCLC patients through the use of multiple screening phases. ANRIL dysregulation may be a significant factor in the development of NSCLC (Hu et al. 2016).

### BC200 RNA

Many human tumors exhibit dysregulated neuronal BC200 RNA expression. In these instances, BC200 RNA was only found in malignant lesions; intratumor stromal areas and matching normal tissue from the same individuals showed little to no specific labeling. Non-neural cancers’ production of BC200 RNA may suggest a functional relation between tumor development and/or metastasis (Chen et al. 1997) which could be the case in lung cancer (to be examined).

### DGCR5

DiGeorge syndrome-associated ncRNA (DGCR5) or LINC00037 is downregulated via its interaction with the proximal upstream binding site of repressor element 1-silencing transcription factor (REST) (Johnson et al. 2009).

### FENDRR (FOXF1-AS1)

In lung cancer, Forkhead Box F1 Antisense 1 (FOXF1-AS1) expression was markedly downregulated. By controlling epithelial-mesenchymal transition (EMT), FOXF1-AS1 overexpression prevents lung cancer cells from migrating and invading. Downregulation of FOXF1 in lung cancer is also associated with loss of FOXF1-AS1 (Miao et al. 2016).

### HIF1A-AS1

Hypoxia inducible factor 1 subunit alpha-antisense 1 (HIF1A-AS1) level were markedly elevated in tumor tissues or serum from patients with NSCLC. Their serum levels were considerably lower following surgery than they were before. These findings showed that elevated blood levels of X-inactive specific transcript (XIST) and HIF1A-AS1 could be employed for the screening of NSCLC (Tantai et al. 2015).

### HMlincRNA717

Compared to nearby non-tumor tissues, the HMlincRNA717 expression level was considerably lower in NSCLC tissues. Its expression was linked to both lymph node metastasis and the histological grade. HMlincRNA717 was demonstrated as an independent prognostic factor for patients with NSCLC by multivariate survival analysis (Xie et al. 2014).

### HOTTIP

The HoxA locus contains a lincRNA called HoxA distal transcript antisense RNA (HOTTIP), which interacts with the mixed lineage leukemia (MLL) complex and the transcriptional activator WD repeat-containing protein 5 (WDR5) which is which is involved in histone methylation. In order to promote gene expression, HOTTIP enables chromatin looping, which recruits the two regulatory factors to the HoxA locus (Giles et al. 2015). Increased levels of HOTTIP may serve as a diagnostic biomarker for sepsis-associated acute respiratory distress syndrome (ARDS) and potentially forecast short-term mortality risk. This can be accomplished by the miR-574-5p sponge (Shi et al. 2024). As an oncogene, HOTTIP regulates the production of Homeobox Protein A3 (HOXA3), which in turn increases cell proliferation and invasion in NSCLC (Su et al. 2022).

### LINC00001 (XIST)

XIST was overexpressed in NSCLC. XIST knockdown suppressed the tumorigenicity of NSCLC cells in vivo and decreased their migration, invasion, and proliferation in vitro. Mechanistically, XIST and Enhancer of zeste homolog 2 (EZH2) may work together to inhibit the transcription of Krüppel-like Factor 2 (KLF2) (Fang et al. 2016).

### LINC00004 (BCYRN1)

In NSCLC, MYC targets and upregulates Brain Cytoplasmic RNA 1 (BCYRN1) which increases cell motility and invasiveness. MYC and BCYRN1 expressions were found to be positively correlated (Hu and Lu 2015).

### LINC00008 (H19)

H19 or LINC00008 an imprinted lncRNA derived from the maternal allele and located within a gene cluster that includes insulin-like growth factor 2 (IGF2).

The expression of H19 is regulated by the GLI1 transcription factor, which facilitates sonic hedgehog (SHH) signaling (Qureshi et al. 2010). H19 transcription is positively influenced by the cell cycle regulatory protein E2F Transcription Factor 1 (E2F1) during the S-phase of proliferating cells (Qureshi et al. 2010).

H19 suppresses NSCLC and is crucial for its invasion and migration. More significantly, H19 may control NSCLC metastasis by altering proteins involved in the cellular signaling system. This is linked to cell adhesion and proliferation, such as epidermal growth factor receptor (EGFR), Metastasis Associated with Colon Cancer 1 (MACC1), β-catenin, and Extracellular signal-regulated kinase 1 and 2 (ERK1/2) (Wang et al. 2016a, b).

H19 single nucleotide polymorphisms (SNPs) were significantly associated with both responsiveness to platinum-based chemotherapy and the risk of developing lung cancer. These SNPs might be useful clinical indicators to forecast a patient’s chance of developing lung cancer and how they will react to platinum-based treatment (Gong et al. 2016a).

The suppression of H19 and miR-107 lead to a significant decrease in cells’ number in the G2/M phase, indicating that H19, triggered by MYC, is up-regulated in NSCLC (Cui et al. 2015; Zhang et al. 2016). Higher H19 expression was positively associated with both tumor size and higher TNM stage. Therefore, H19 plays a role in NSCLC oncogenesis. Additionally, H19 may be a target for new therapeutics and a possible diagnostic indicator in NSCLC patients (Zhang et al. 2016).

H19 SNP rs2067051 is associated with a lower incidence of coal workers pneumoconiosis. This finding could offer a novel genetic marker for early detection and screening of pneumoconiosis in high-risk populations (Wu et al. 2016a).

### LINC00023 (MEG3)

It was found that maternally expressed gene 3 (MEG3) or LINC00023 expression was markedly elevated in lung adenocarcinoma (LAD). Therefore, suggesting that MEG3 plays a carcinogenic role. MEG3, as a competing endogenous RNA (ceRNA) that may down-regulate miR-106. This is followed by up-regulating Mitogen-activated protein kinase 9 (MAPK9) to promote the development of lung cancer (Li et al. 2016). In another study, through the regulation of p53 and B-cell lymphoma-extra-large (Bcl-xl) expression, MEG3 is dramatically downregulated in LAD. MEG3, additionally, partially controls the cisplatin resistance of LAD cells. As a result, MEG3 might be a novel indicator of a poor response to cisplatin and a possible target for LAD treatment (Liu et al. 2015).

In NSCLC specimens, S-phase kinase-associated protein 2 (Skp2) levels were significantly higher. Therefore, in order to prevent NSCLC cell proliferation, according to the study by Su, Han et al. in 2016, MEG3 and miR-3163 may work together to reduce Skp2 mRNA translation in NSCLC cells (Su et al. 2016).

### LINC00047 (MALAT-1)

The metastasis-associated lung adenocarcinoma transcript 1 (MALAT-1) is known as LINC00047.

MALAT-1 and colon cancer-associated transcript 2 (CCAT2) genetic polymorphisms could predict the risk of lung cancer and the response to platinum-based chemotherapy. This could be attributed to being significantly associated with both lung cancer susceptibility and platinum-based chemotherapy response (Gong et al. 2016a). The transcription factor Sp1 facilitated the overexpression of MALAT-1 in lung cancer cells, suggesting that Sp1 could be a potential cancer treatment target (Li et al. 2015a, b).

The highly invasive subline of lung cancer cells that metastasize to the brain has elevated MALAT-1. Therefore, MALAT-1 silencing prevents the migration and metastasis of highly invasive subline of lung cancer cells to the brain by triggering the EMT (Shen et al. 2015).

MALAT-1 was found to be a predictive factor for patient survival in stage I NSCLC by Kaplan–Meier analysis which aids in the identification of early-stage NSCLC patients who are very susceptible to metastasis (Ji et al. 2003). MALAT-1 may be also able to predict the tumor outcome (Rotblat et al. 2011). The development and proliferation of the tumor tissue in NSCLC xenografts were hampered by reduced MALAT-1 expression. On the genetic level, MALAT-1 has the strongest correlation with genes related to immunological regulation, cellular development, motility, proliferation, and signaling that are implicated in cancer (Schmidt et al. 2011a).

MALAT-1 risk scores were associated with the progression of NSCLC and its diagnostic efficacy for stages I, II, and III was comparatively high, suggesting that it may be a useful tool for early NSCLC detection (Peng et al. 2016).

Compared to normal lung cells or tissues, lung cancer cells or tissues have fewer methylated forms of MALAT-1 promoter revealing that DNA methylation is necessary for MALAT-1 expression (Guo et al. 2015a, 2015b, 2015c). In localized NSCLC, Bcl-2 expression was particularly linked to a better prognosis. It was discovered that Bcl-2 and MALAT-1 expression interact, which is worth looking into further for risk prediction in patients with resectable NSCLC (Schmidt et al. 2014).

A strong correlation was discovered between the risk of PAH and the genomic variations in MALAT-1 loci. The risk of PAH was lower in carriers with genotypes for the G variation. Additional functional experiments revealed that the genomic variations in MALAT-1 could directly increase the expression of X box-binding protein 1 (XBP1), where MALAT-1 to act as ceRNA competing with miR-214. This, in turn, could suppress the migration and proliferation of vascular endothelial cells in vitro by shortening the S-M phase transition (Zhuo et al. 2017).

### LINC00057 (SNHG1)

Compared to normal bronchial epithelial cells, lung cancer cells exhibited a substantial upregulation of the lincRNA Small nucleolar RNA host gene 1 (SNHG1) expression. Furthermore, in vitro tests showed that SNHG1 knockdown reduced cell division (You et al. 2014).

### LINC00064 (HAR1)

LINC00064 known as human accelerated region 1 or HAR1 which is co-expressed in Cajal-Retzius cells of the human neocortex along with the crucial neural factor Reelin (RELN), guiding developmental mechanisms. HAR1 is also expressed in the developing neurons of the human embryonic neocortex and is regulated by the REST through DNA-transcription factor (TF) interactions (Johnson et al. 2010).

### LINC00066 (MIAT)

Myocardial infarction-associated transcript (MIAT) lincRNA is linked to tumor proliferation. MIAT may be crucial in the emergence of LAD through their interactions with hsa-mir-106. The later controls MAPK9 to participate in MAPK signaling pathways (Li et al. 2016). MIAT may work as a ceRNA to control lung cancer cell invasion, migration, and proliferation by forming a feedback loop involving MAPK9 and miR-106 (Li et al. 2016).

### LINC00079 (PVT-1)

The Plasmacytoma Variant Translocation 1 (PVT-1) is involved in cellular proliferation through DNA-TF regulation of MYC. PVT-1 acts as a downstream target of MYC (Carramusa et al. 2007). A previous study found that the PVT-1 rs378854 (G) allele reduces the binding of the TF Yin Yang (YY1) in vitro (Meyer et al. 2011).

Additionally, lung squamous cell carcinoma patients (LSCC) proliferation was suppressed by PVT-1 knockdown (Wei and Zhang 2016).

A greater TNM stage, a larger tumor, and a lower OS were all attributed to the upregulation of PVT-1 in human NSCLC tissues. By controlling the Mdm2-p53 pathway, ectopic expression of LATS2 suppressed LAD cell growth and promoted apoptosis (Wan et al. 2016a).

### LINC00080 (TUG-1)

Taurine-upregulated gene 1 (TUG-1) recruits and binds to PRC2, is a direct transcriptional target of p53 and is typically downregulated in NSCLC tissues. Expression of Homeobox B7 (HOXB7) may be increased by TUG-1 inhibition, via engaging in the AKT and MAPK pathways (Zhang et al. 2014). TUG-1 RNA could bind to PRC2 in the promotor region of CUGBP and Elav-like family member 1 (*CELF1*) and suppress *CELF1* production in NSCLC cells (Lin et al. 2016).

### LINC00084 (NEAT1)

LINC00084 or the nuclear enriched abundant transcript 1 or nuclear paraspeckle assembly transcript 1 (NEAT1) dramatically speeds up the formation of tumors in vivo and in vitro NSCLC cell growth and metastasis. As a ceRNA for hsa-miR-377-3p, NEAT1 counteracted its actions and caused its endogenous target, E2F transcription factor 3 (E2F3) de-repression (Sun et al. 2016). By sponging hsa-mir-98-5p in NSCLC, NEAT1 functions as a ceRNA to upregulate the green tea polyphenol EGCG-induced copper transporter 1 (CTR1) (Jiang et al. 2016).

### LINC00115

LINC00115 is an oncogenic lncRNA located on the short arm of chromosome number 1 (1p36.33), implicated in lung cancer progression. Linc00115 downregulation offers potential as a therapeutic target to inhibit tumor growth and metastasis (Xu and Nemati 2024). *Shao *et al*.* in 2021 claimed the expression of LINC00115 altered the expression of EMT-related proteins, promoting E-cadherin while inhibiting N-cadherin, vimentin, and fibronectin (Shao et al. 2021a).

In addition, through competitive interactions with miR-7, LINC00115 may contribute to LAD by first downregulating hsa-miR-7 and then upregulating Fibroblast growth factor 2 (FGF2) (Li et al. 2016).

### LINC00173

High levels of LINC00173 correlate with advanced disease stages and poor prognosis in SCLC patients and are linked to shorter survival rates. MiR-218 often acts as a tumor suppressor, and its downregulation is frequently associated with poor prognosis. In SCLC, miR-218 is often found to be downregulated, particularly in chemoresistant cell lines and tissues. This loss of function contributes to tumor progression and resistance to chemotherapy (Yang et al. 2017).

Epithelial Tyrosine Kinase (Etk) is a non-receptor tyrosine kinase involved in various signaling pathways that regulate cell growth, survival, and motility (Wang et al. 2018). This interaction prevents miR-218 from targeting and downregulating Etk, leading to increased Etk expression (Zeng et al. 2020).

### LINC00178 (UCA1)

While urothelial cancer associated 1 (UCA1) knockdown hindered the growth and colony formation of NSCLC cells, UCA1 overexpression improved these processes. Furthermore, mechanistic studies demonstrated that UCA1 competitively "sponged" miR-193a-3p to increase the expression of the miR-193a-3p target gene Erb-b2 receptor tyrosine kinase 4 (*ERBB4*) (Nie et al. 2016). Lung cancer cells that had acquired resistance to EGFR-tyrosine kinase inhibitors (TKIs) showed markedly elevated UCA1 expression. In acquired resistant cells with non-T790M, UCA1 knockdown restored gefitinib sensitivity and inhibited the activation of EMT and AKT/mTOR pathway (Yang et al. 2014; Cheng et al. 2015).

### LINC00346

LINC00346 exhibited upregulation in LUAD tissues and cells, primarily concentrated in the cytoplasm. Knockdown of LINC00346 suppressed in vivo tumor growth, proliferation, metastasis, and cell cycle progression while promoting apoptosis. LINC00346 sequestered miR-30c-2–3 by targeting MYBL2 and modulating the cell cycle signaling pathway. Inhibiting miR-30c-2-3p or overexpressing MYBL2 could counteract the suppressive impact of LINC00346 knockdown on the progression of LUAD. Thereby, LINC00346 functions as a ceRNA, contributing to the carcinogenesis of LUAD through the miR-30c-2-3p/MYBL2 axis, which regulates the cell cycle signaling pathway (Xu et al. 2021).

### LINC00483

LINC00483 shows upregulation in LAD tissues and cell lines. Elevated LINC00483 levels are strongly associated with reduced survival durations, advanced TNM staging, increased tumor size, and positive lymph node metastases. Cell proliferation, migration, and invasion were inhibited following the knockdown of LINC00483. LINC00483 is mostly localized in the cytoplasm, functioning as a sponge for miR-204-3p. ETS1 was confirmed as a downstream target of miR-204-3p and is consequently controlled by LINC00483 (Yang et al. 2019a, b).

### LINC00511

In a clinical trial done on LAD and LSCC, the LINC00511 was found to be upregulated in LAD tissues compared to normal ones, and the knockdown of LINC00551 resulted in impaired proliferation of LAD cells (Wei and Zhang 2016).

### LINC00520

The expression of LINC00520 was raised in LUAD tissues and cell lines. The expression of LINC00520 exhibited an inverse correlation with the level of miR-1252-5p. FOXR2 is a target of miR-1252-5p; hence, LINC00520 may function as a ceRNA to regulate FOXR2 levels by sequestering miR-1252-5p, thereby serving for LUAD treatment monitoring (Chen et al. 2021).

### LINC00586 (BANCR)

LINC00586 or BRAF-activated non-coding RNA (BANCR) expression knockdown is linked to higher tumor size. In Lewis lung cancer cells, it was found that histone deacetylation played a role in controlling BANCR (Chen et al. 2015). Activation of p38 MAPK and JNK was reduced by BANCR overexpression. BANCR silencing saved the inactivation of p38 MAPK and JNK to contribute to the migration and proliferation of lung cancer (Jiang et al. 2015).

Significantly lower levels of BANCR expression were found in NSCLC tumor tissues. Moreover, BANCR is associated with larger tumors, more advanced clinical stages, further metastases, and a lower OS rate for NSCLC patients. The downregulation of BANCR in NSCLC cells was attributed to histone deacetylation. BANCR overexpression regulates the production of E-cadherin, N-cadherin, and vimentin, which is important for EMT (Sun et al. 2014).

### LINC00673

The ectopic expression of LINC00673 enhances the motility and invasion of NSCLC cells. LINC00673 could suppress HOXA5 expression by binding the epigenetic repressor EZH2 at its promoter regions. HOXA5 has been recognized as a tumor suppressor gene that inhibits the metastasis of NSCLC cells via modulating cytoskeletal reorganization. The up-regulation of LINC00673 expression transcriptionally inhibited target gene expression in NSCLC cells by directly binding to RNA-binding proteins, hence promoting cancer cell proliferation and dissemination (Ma et al. 2017).

### LINC00873 (CCAT2)

NSCLC tissues showed also a significant overexpression of CCAT2. Interestingly, silencing CCAT2 with small interfering RNA (siRNA) inhibited invasion and proliferation in NSCLC cell lines in vitro, and CCAT2 over-expression was substantially associated with LAD but not LSCC. Furthermore, CCAT2 and carcinoembryonic antigen (CEA) together may be able to predict the lymph nodes metastasis (Qiu et al. 2014).

In vitro, NSCLC cell lines’ proliferation and invasion were inhibited when CCAT2 was silenced by siRNA. Additionally, lymph node metastases could be predicted by CCAT2 when combined with CEA (Qiu et al. 2014).

The expression of lncRNA CCAT2 was increased in SCLC cell lines and tissues, and it was linked to the patient’s poor prognosis and malignant state. Moreover, SCLC cell growth and metastasis were successfully inhibited in vitro by CCAT2 expression suppression (Chen et al. 2016).

### LINC00902 (TUSC7)

Higher TNM stage and greater tumor size have been correlated to lower Tumor suppressor candidate 7 (TUSC7) expression in NSCLC tissues. When compared with patients with high expression, those with reduced TUSC7 expression had a poorer OS rate. Downregulation of TUSC7 was proposed as an independent poor prognostic factor for patients with NSCLC. Furthermore, in vitro lung cancer cell proliferation may be inhibited by upregulating TUSC7 expression (Wang et al. 2016a, b).

### LINC00973

The expression of LINC00973 in NSCLC tissues was elevated, and this higher expression correlated with the poor prognosis of NSCLC patients, leading to the construction of the LINC00973-miRNA-mRNA ceRNA network competition mechanism. The majority of miRNA and mRNA inside the competitive ceRNA network have been validated to play significant biological functions in the advancement of NSCLC (Guo et al. 2021).

### LINC01031

Lung squamous cell carcinoma (LSqCC) exhibited a unique transcriptional profile of LINCRNAs when contrasted with normal lung tissues. Certain lincRNAs may accurately forecast lung cancers. In LSqCC patients, elevated levels of LINC01031, LINC01088, and LINC01931 were substantially correlated with unfavorable prognosis, indicating potential targets for anti-LSqCC therapy (Liu et al. 2019). The previous lincRNAs may facilitate LSqCC via their adjacent genes. LINC01031 is situated at 1q31.2, in proximity to the protein-coding genes B3GALT2 (β−1,3-Galactosyltransferase 2), CDC73 (cell division cycle 73) (Walls et al. 2017), Grx2 (glutaredoxin-2) (Webb et al. 1989; Lundberg et al. 2001), and F13B (coagulation factor XIII B component).

### LINC01089

The expression of LINC01089 was markedly downregulated in LUAD tissues and cells, and its overexpression diminished cell proliferation and migration. LINC01089 modulates STARD13 expression by competitively binding to miR-301b-3p in LUAD. Furthermore, the depletion of STARD13 or the overexpression of miR-301b-3p reverses the inhibitory effect of LINC01089 knockdown on the phenotypes of LUAD cells. Thus, LINC01089 functions as a tumor inhibitor in LUAD by targeting the miR-301b-3p/STARD13 axis, offering a potential perspective on LUAD therapy (Qian et al. 2021).

### LINC01133

A previous study revealed that although it was not present in the LAD samples, LINC01133 was elevated in LSCC. Patients with LSCC who expressed more LINC01133 had shorter survival time. Transwell and wound-healing experiments showed that LSCC cell line invasion was decreased when LINC01133 was silenced by siRNA (Zhang et al. 2015).

### LINC01138

Additionally, the knockdown of LINC01138 reduced fluctuation, proliferation, and migration in A549 and H460 LUAD cells. LINC01138 provides a prognostic classifier with strong predictive performance (Xiao et al. 2022).

### LINC01511 (RP11-325I22.2)

According to a previous study, lung cancer EGFR exon 19 deletions resulted in aberrant expression of several lincRNAs. The EGFR exon 19 deletions in lung cancer were substantially greater than wild-type EGFR, and the RP11-325I22.2 was most markedly elevated. According to this finding, RP11-325I22.2 may be crucial to the process behind EGFR exon 19 deletions in lung cancer (Wang et al. 2014).

### LINC01627 (RP11-397D12.4), LINC01628 (AC007403.1), and ERICH1-AS1

Three circulating lncRNAs, RP11-397D12.4, AC007403.1, and ERICH1-AS1 were found in a prior study to function as predictors for NSCLC and to be putative fingerprints for predicting carcinogenesis (Tang et al. 2015).

### LINC01833

LINC01833 can markedly enhance the proliferation and invasive capacity of LAUD cells and facilitate the EMT process. LINC01833 can act as a competitive ceRNA by sequestering miR-519e-3p through a sponge mechanism. S100A4 is a direct target of miR-519e-3p. Consequently, LINC01833 influenced the miR-519e-3p/S100A4 axis in LAUD (Zhang et al. 2020b).

### LINC-MD1

LINC-MD1 yields miR-206 in one intron and miR-133b in one exon. Previous research has connected MiR-206 to vascular remodeling in PAH (Thum and Dimmeler 2017). Overexpression of miR-206 also downregulates Notch 3, a critical mediator of PAH development. Per, Notch-associated lincRNAs profiling circuits epigenetic modification(s), this could be the case for the lung (a point to be examined). Therefore, miR-206 regulates vascular remodeling in PAH via several mechanisms that are important in disease pathology (smooth muscle cell proliferation, apoptosis, and differentiation), suggesting that it may improve pulmonary arterial SMC therapy.

Although there is no causal relation that has been shown between PAH and miR-133b in the lung, however, it is a biomarker of right ventricular hypertrophy and thus PAH. Furthermore, LINC-MD1 has binding sites for miR-133. LINC-MD1 is exclusively situated in the cytosol of cells, consequently, its modulation could be advantageous in the pathophysiology of PAH (Ballantyne et al. 2016).

### LINC-PINT

The long intergenic non-protein coding RNA, p53-induced transcript (LINC-PINT) gene is located on chromosome 7 long arm (7q32.3). LINC-PINT functions as a ceRNA for miR-208-3p, thereby upregulating programmed cell death protein 4 (PDCD4) and diminishing the proliferation and cell-cycle progression of NSCLC (Zhang et al. 2019a, b). LINC-PINT upregulation promoting LUAD-associated transcript-1 (UPLA1) exhibited elevated expression in the nucleus of lung adenocarcinoma (LAD) cells, markedly enhancing tumor growth via triggering the Wnt/β-catenin signaling pathway (Han et al. 2020). Its downregulation correlates with tumor aggressiveness and poorer patient survival. LINC-PINT represses a pro-invasion gene signature, including genes regulated by the transcription factor, early growth response‐1 (EGR-1). It mediates this repression through PRC2, enhancing its binding to target gene promoters. LINC-PINT interacts with PRC2, influencing epigenetic modification through tri-methylation of lysine 27 on histone H3 protein (H3K27me3) that silences invasion-related genes (Marín-Béjar et al. 2017). LINC-PINT enhances PTEN expression by sponging miR-543, counteracting its repressive effects (Wang et al. 2020a, 2020b, 2020c).

### LINC-RoR

LincRNA-regulator of reprogramming (LINC-ROR) was shown to be directly regulated by the well-known three pluripotent factors Oct4, Sox2, and Nanog (Chen et al. 2023) exerting DNA-DNA-TF interaction through co-localization of close to its promoter region (Loewer et al. 2010).

LINC-ROR was significantly upregulated in NSCLC tissues compared to adjacent normal tissues. Higher levels of LINC-ROR are associated with advanced TNM stage, positive distant metastasis, and lymph node metastasis. Patients with elevated LINC-ROR expression have poorer OS and disease-free survival (DFS), with statistical analyses indicating that LINC-ROR is an independent prognostic factor for both OS and DFS (Qu et al. 2017).

LINC-ROR is significantly elevated in docetaxel-resistant LAD cells, contributing to their chemoresistance and mesenchymal characteristics. Reducing LINC-ROR levels reverses EMT and enhances sensitivity to docetaxel. LINC-ROR acts as a ceRNA for miR-145, inhibiting its function and leading to increased expression of its target, Fascin Actin-Bundling Protein 1 (FSCN1), which is associated with drug resistance and EMT (Pan et al. 2017).

### LincRNA-COX2

In acute lung injury, lincCOX2 modulates oxidative stress through the Nrf2/ARE signaling pathway meanwhile its downregulation mitigates oxidative stress through the Nrf2/ARE pathway. Therefore, lincCOX2 could serve as a viable target for acute lung injury treatment (Xie et al. 2024). LincRNA-COX2 plays a regulatory role in the progression of PAH. lincRNA-COX2 was elevated in peripheral blood samples from individuals with PAH and in hypoxic pulmonary artery smooth muscle cells (PASMCs) in a time-dependent manner. The miR-let-7a/STAT3 axis, demonstrated to be crucial in the development of PAH, was controlled by lincRNA-COX2. Consequently, lincRNA-COX2 may modulate the progression of PAH via the miR-let-7a/STAT3 pathway, which holds substantial importance for the investigation of targeted therapies for PAH (Cheng et al. 2020).

### LincRNA-p21

LincRNA-p21 promotes lung fibroblast proliferation in ARDS by suppressing the expression of thymocyte differentiation antigen-1 (Thy-1). The increased collagen levels in lung and bronchoalveolar lavage generated by lipopolysaccharide (LPS) could be mitigated by lincRNA-p21 interference. Hence, lincRNA-p21 is a possible diagnostic biomarker and a likely contributor to the etiology of pulmonary fibrosis in acute lung injuries, including ARDS (Wang et al. 2016a, b). Inhibition of lincRNA-p21 demonstrated an adverse effect on LPS-induced p65 nuclear translocation and the enhancement of NF-κB activity. lincRNA-p21 inhibition could alleviate LPS-induced lung injuries in vivo. Consequently, lincRNA-p21 may play a significant role in the LPS-induced pro-inflammatory response through NF-κB/p65 mediated pathways, indicating its potential application in ARDS therapy (Zhang et al. 2020a, b). lincRNA-p21 (also known as tumor protein p53 pathway core pressor 1) was shown to inhibit the translation of target mRNAs by negatively regulating the translation of CTNNB1 (β-catenin) and JUNB (transcription factor jun-B) (Yoon et al. 2012).

### LncRNA-ATB

Pneumoconiosis in coal miners is also associated with elevated lncRNA-activated by TGF-β (lncRNA-ATB) expression, which is substantially correlated with TGF-β1 in these patients. Additionally, increased lncRNA-ATB was linked to a higher incidence of pneumoconiosis among coal miners (Cui et al. 2016).

### LnRPT

LnRPT (lncRNA controlled by PDGF and transforming growth factor β) was identified as the most effective in enhancing the proliferation of PASMCs upon knockdown. The overexpression of LnRPT inhibited the growth of PASMCs. LnRPT mechanistically suppressed the expression of two genes associated with the Notch signaling system (notch3 and jag1) and the cell-cycle regulatory gene ccna2. The downregulation of LnRPT produced by PDGF-BB was negated when phosphatidylinositol 3′-kinase activity was suppressed using pictilisib. Downregulation of LnRPT was also noted in the pulmonary arteries of rats with MCT-induced PAH. Thus, LnRPT functions as a regulator of PASMC proliferation in the development of PAH PAH by modulating the Notch signaling pathway and the cell cycle (Chen et al. 2018).

### PAXIP1‐AS1

PAXIP1‐AS1 plays a fundamental function in regulating the hyperproliferative and migratory behaviors of SMCs in idiopathic PAH. Additionally, PAXIP1‐AS1 mechanistically disrupts the focal adhesion axis by modulating the expression and phosphorylation of its downstream target, paxillin (Jandl et al. 2019). Under hypoxic circumstances, Hoxaas3 was up-regulated in PASMCs and the pulmonary vasculature of hypoxic mice. The activation of Hoxaas3 gene expression was enhanced by histone H3 lysine 9 acetylation. Additionally, through up-regulating Homeobox a3 at the mRNA and protein levels, increased expression of Hoxaas3 was linked to cell proliferation and altered cell cycle distribution (Zhang et al. 2019a, b).

### SNHG5 lncRNA

LncRNA SNHG5 and miR-16–2-3p are intricately associated with the onset and prognosis of rare-earth pneumoconiosis (REP) via inflammatory responses, potentially serving as biomarkers for REP. SNHG5 is implicated in the p38/MAPK signaling pathway, Wnt/CTNNB1, and other signaling pathways associated with pulmonary fibrosis (Chen et al. 2018).

### SPRY4-IT1

Through the control of E-cadherin and vimentin expression, upregulated Sprouty RTK signaling antagonist 4-intronic transcript 1 (SPRY4-IT1)in NSCLC tumor tissues has been found to be a predictor for poor prognosis of NSCLC by promoting EMT (Hu et al. 2016).

**Fig. 1:**
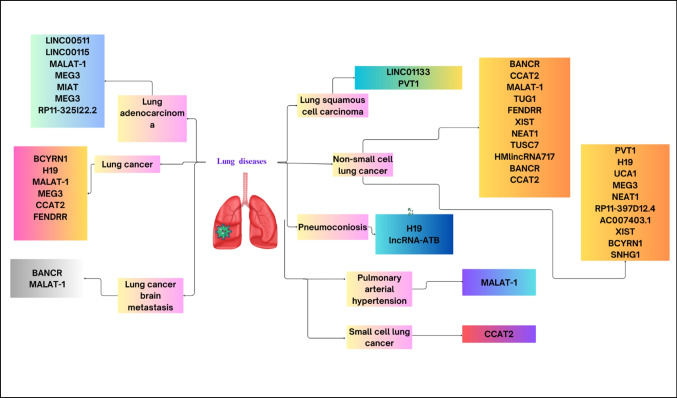
Human lincRNAs in different lung diseases

Figure 1 is dictating the previously mentioned lincRNAs in various lung diseases.

[MALAT-1: Metastasis associated lung adenocarcinoma transcript 1; NEAT2: Nuclear-enriched abundant transcript 2; MEG3: Maternally expressed gene 3; MIAT: Myocardial infarction associated transcript; EGFR: Epidermal growth factor receptor; BCYRN1: Brain Cytoplasmic RNA 1; CCAT2: Colon cancer-associated transcript 2; FENDRR: Fetal-lethal non-coding developmental regulatory RNA; BANCR: BRAF activated non-coding RNAs; MAPK: Mitogen-activated protein kinase; PVT-1: Plasmacytoma Variant Translocation 1; TDB 43: TAR-DNA-binding Protein 43; TUG-1: Taurine-upregulated gene 1; HOXB7: Homeobox B7; CELF1: CUGBP and Elav-like family member 1; XIST: X inactive specific transcript; KLF2: Krüppel-like Factor 2; NEAT1: Nuclear enriched abundant transcript 1; EGCG: Epigallocatechin-3-gallate; CTR1: Copper transporter 1; TUSC7: Tumor suppressor candidate 7; EZH2: Enhancer of zeste homolog 2; LATS2: Large tumor suppressor kinase 2; UCA1: Urothelial cancer associated 1; *ERBB4*: Erb-b2 receptor tyrosine kinase 4; TKIs: Tyrosine kinase inhibitors; *SKP2*: S-phase kinase-associated protein 2; SNHG1: Small nucleolar RNA.]

## Diagnostic and prognostic lincRNAs in lung cancer

Single serum markers like cytokeratin fragment antigen 21–1 (CYFRA21-1), CEA, and CA125 have little diagnostic utility for lung cancer (Yang-Chun et al. 2016).

**Fig. 2:**
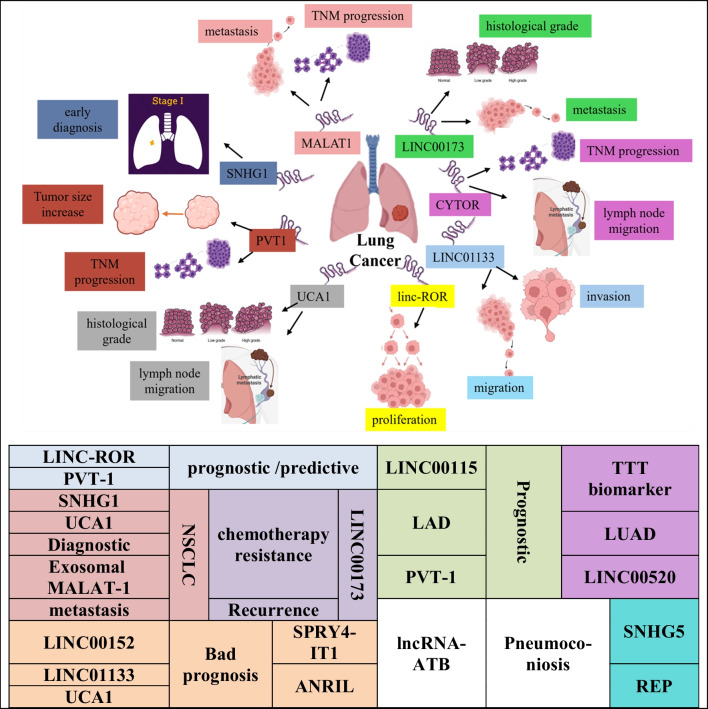
LincRNAs involved in lung cancer diagnosis and prognosis

Apart from their significant involvement in lung cancer growth and metastasis, lincRNAs also hold significant clinical importance (Fig. 2). LincRNAs exhibit notable stability in body fluids as well, even after repeated freezing and thawing, a 24-h incubation at 45 °C, a 24-h incubation at room temperature, and other extreme circumstances (Arita et al. 2013).

**Exosomal ncRNAs** represent a promising milieu for diagnosis or prognosis nowadays in various cancer types (Hamdy et al. 2024a, b, c, d; Rizk et al. 2024).

**Exosomal** **MALAT-1** serum expression level may be utilized as a diagnostic biomarker for NSCLC metastasis, according to Zhang et al.’s findings (Zhang et al. 2017a, b).

Investigating the clinicopathological parameters revealed a correlation between high **LINC00173** expression, tumor metastases, and tumor histological type. Furthermore, LINC00173 levels were reduced during chemotherapy, but increased again upon recurrence, suggesting its role in chemotherapy resistance (Yang et al. 2020a, b).

A predictive factor for NSCLC patients may be the high expression of cytoskeleton regulator RNA (CYTOR or **LINC00152**) in NSCLC tissues (Zhang and Li 2018). Additionally, in NSCLC tissues, **LINC01133** is significantly overexpressed. This overexpression may play a crucial role in the course of NSCLC and is associated with a bad prognosis for the patient (Zang et al. 2016).

One of the potential prognostic and predictive biomarkers for NSCLC is the high expression of **LINC-ROR** which promotes tumor growth and cell proliferation (Qu et al. 2017). Xia et al. showed that ROR was overexpressed in both NSCLC and LCSCs and that a poor prognosis in NSCLC was associated with high ROR expression (Wang et al. 2016a, b). \According to Wan et al. **PVT-1** is elevated in LAD tissues and this upregulation was linked to higher pathological stage and tumor size. Crucially, the poor prognosis may be associated with the overexpression of PVT-1, which may also function as an independent prognostic indicator (Wan et al. 2016a).

According to Zhu et al. lung cancer tissues exhibit significantly higher levels of lncRNA16 or **SNHG1** expression than adjacent non-cancer tissues. Also, patients with early-stage lung cancer had greater levels of lncRNA16 (SNHG1) expression in their plasma that might be a useful biomarker for lung cancer early diagnosis (Zhu et al. 2017).

According to Wang et al. histological grade and lymph node migration were linked to **UCA1** overexpression in the plasma of NSCLC patients, which may serve as a biomarker for NSCLC diagnosis and an indicator of poor survival rates (Wang et al. 2015).

**LINC00115** may be a prognostic biomarker in LAD tumorigenesis (Li et al. 2016). Upregulated **SPRY4-IT1 and ANRIL** in NSCLC tumor tissues has been found to be a biomarker for a poor prognosis of NSCLC (Pan et al. 2015; Hu et al. 2016).

**XIST and HIF1A-AS1** could be employed as predictive biomarkers for the screening of NSCLC (Tantai et al. 2015). Additionally, increased **lncRNA-ATB** was linked to a higher incidence of pneumoconiosis and could be a biomarker for the disease among coal miners (Ma, Cui et al. 2016).

**LINC00520** may serve as an effective biomarker for LUAD treatment (Chen et al. 2021), and **SNHG5** potentially could serve as a biomarker for REP (Chen et al. 2018).

Moreover, the potential of lincRNAs as lung cancer diagnostic markers should be further clarified. Combining ncRNA-based diagnostics or treatments with chemotherapy, immunotherapy, or existing biologics for asthma or COPD should be further investigated.

Different lincRNAs can be used as diagnostic and/or prognostic biomarkers for lung cancer as they are associated with different pathological conditions. MALAT-1 is strongly associated with tumor stage and lymphatic metastasis. LINC00173 expression is correlated with tumor metastases and tumor histological type. CYTOR is associated with poor prognosis, lymph node migration, and progressing TNM stage. LINC01133 high expression is related to tumor invasion, migration, and poor prognosis. LINC-ROR is linked to tumor growth and cell proliferation. PVT-1 was associated with higher pathological stage and tumor size. SNHG1 enhanced the G2/M transition by regulating cyclin B1 production in a manner that is independent of p53 suggesting its potential use as an early diagnostic biomarker. UCA1 is associated with histological grade and lymph node migration and can be used as an indicator of poor survival rates.

## Drugs against some lincRNAs in lung cancer

The logical approach to drug development has superseded the empirical one, allowing tailored medicines to target the aberrant dominant mutation, gene amplification, or oncogenic translocation that supports tumor growth. Few solid cancers evolve by hyperactivating targeted genes, a hallmark of targeted treatment. Due to the molecular analysis showing that intrinsic resistance patients lack drivers, targeted treatment will not be started (Ellis and Hicklin 2009).

Nano-biomedicine targeting ncRNAs could be a promising precision therapeutic approach (Hamdy et al. 2022a, b; Hamdy et al. 2022a, b; Hamdy et al. 2024a, b, c, d; Sokolov et al. 2024).

This section will shed the lights toward ncRNAs for lung cancer therapy, specific lncRNA targeting therapies and nanoparticles targeting lncRNAs.

The benefits of implementing ncRNAs for lung cancer therapy have been assessed widely in preclinical models. Compared to siRNAs, miRNAs display imperfect complementarity to mRNAs such that it has the unique ability to affect multiple genes with fewer molecules required (Laganà et al. 2014; Le et al. 2021). Based on the previously mentioned, lincRNAs that suppress malignancies may be restored through the use of gene therapy or synthetic RNA molecules. Carcinogenic lincRNAs may be halted by small compounds. Quercetin, a small molecule that was recently discovered, bonds to a MALAT-1 triplex, thereby affecting the levels and functions of MALAT-1 transcripts in vitro (Rakheja et al. 2022). Another minor chemical that was recently identified, AC1NOD4Q, restricts HOTAIR’s connection to EZH2 (a PRC2 subunit), thereby inhibiting downstream target methylation (Wang et al. 2019a, b). CRISPR-based methods are innovative methods for silencing lincRNAs in pre-clinical animals (Tontonoz et al. 2017).

The clinical application of lincRNA targeting techniques is problematic due to the limited sequence conservation between humans and animals. The MALAT-1 gene is a viable candidate for therapeutic target research due to its high degree of conservation across species (Ma et al. 2015). lincRNAs are less likely to cause unwanted adverse effects when administered therapeutically due to their tissue-specificity. Genetically engineered mice, xenografts, organoids, and patient-derived cell lines are all preclinical models for MALAT-1 targeting. Compared to non-targeting controls, subcutaneous MALAT-1 ASO reduced metastasis and increased cancer cell differentiation in a MMTV-PyMT breast cancer mouse model. MALAT-1 ASOs were found to inhibit branching morphogenesis in a three-dimensional breast cancer organoid model. Nude rodents that were administered MALAT-1 ASO systemically exhibited significantly lower lung cancer cell colonization from patients than non-targeting controls (Arun et al. 2016).

One of the new therapeutic strategies in lung cancer is nanoparticles. Nanoparticles and tumor-suppressing or oncogenic RNAs are viable options for targeting lincRNAs in the modern era. Nanoparticle-based delivery systems can enhance the targeting of lincRNAs by providing a means to deliver RNA therapeutics specifically to cancer cells. For example, studies have demonstrated that nanoparticles can be engineered with ligands that bind selectively to receptors overexpressed on cancer cells, facilitating the precise delivery of lncRNA-targeting agents. This active targeting approach not only improves the localization of therapeutic agents but also enhances their uptake by cancer cells, thereby increasing therapeutic efficacy (Awasthi et al. 2019). For examples, Research has identified particular lncRNAs, such as H19 and HOTAIR, that are overexpressed in lung cancer and contribute to resistance against targeted therapies like TKIs. By utilizing nanoparticles to deliver siRNAs targeting these lncRNAs, researchers aim to reverse resistance mechanisms and improve treatment outcomes. Another example, downregulating H19 has been shown to sensitize NSCLC cells to EGFR-TKI therapy, indicating a direct link between lncRNA expression and therapeutic response (Arun et al. 2018; Jiang et al. 2021).

Nano-conjugated MEG3 demonstrated a greater improvement in histopathology and tumor-associated biomarkers when administered intra-hepatically to liver cancer mice than unconjugated MEG3. These factors must be taken into account when determining whether nanoparticles are more effective as interventions, despite the fact that this study did not evaluate blood retention or adverse effects (Elzallat et al. 2023). A second study administered nanoparticle-packaged siRNA to nude rodents with breast cancer, which targeted DANCR, a tumor-promoting lncRNA. The approach inhibited tumor growth, and no alterations in liver, kidney, or lung morphology or histology were observed (Vaidya et al. 2019). The efficacy of this technique in cell lines in reducing migration and invasion and suppressing DANCR suggests that it should be investigated in animal models, despite the fact that it has not been evaluated in in vivo lung cancer models (Nicolescu et al. 2022).

Through searching in databases on lincRNAs expressed in lung cancer with identified target gene locus and drugs active against these lincRNAs. LncRNAWiki 2.0 mentioned 3 lincRNAs could be modulated by different chemical agents as mentioned in Table 3.

**Table 3:** List of individual lincRNAs expressed in lung cancer with identified target gene locus and drugs active against these lincRNAs retrieved from LncRNAWiki 2.0

LincRNA	Target	Gene locus	Drug(s)	Ref
LINC-PINT	-	7q32.3	Curcumin, cisplatin, panobinostat	
				(Marín-Béjar et al. 2017; Wang et al. 2020a, 2020b, 2020c)
LincROR	FSCN1	18q21.31	Tamoxifen, cisplatin, sorafenib, docetaxel, polyphyllinI, gemcitabine	(Pan et al. 2017; Qu et al. 2017; He et al. 2019)
LINC00173	miR-218/ETK	12q24.22	Cisplatin	(Zeng et al. 2020)

https://ngdc.cncb.ac.cn/lncbook/omics/expression Accessed September 27th, 2023.

[FSCN1: Fascin-1 protein; ETK: Transketolase Activation.]

The dysregulated either up or down-regulated, oncogenic or tumor suppressor lincRNA(s) profile observed in lung cancer suggest(s) that these lincRNAs may be considered as potential tumor diagnostic or prognostic markers as well as being a novel next effective therapy targets, a step toward ncRNA precision, to achieve “Better Health” (Sustainable Development Goal #3).

**Linc-p53-induced transcript (LINC-PINT)** was found to be reduced in lung cancer and inhibits lung cancer progression via sponging miR-543 and inducing PTEN. Linc PINT was expected to be targeted by curcumin, cisplatin, panobinostat (Wang et al. 2020a, 2020b, 2020c). While LINC00173 was targeted by cisplatin, lincROR was found to be targeted by Tamoxifen, cisplatin, sorafenib, docetaxel, polyphyllin I, and gemcitabine.

## LincRNA as therapeutic targets

Despite the marked advancement in treating different lung diseases, still, the outcome of the therapeutic strategies is sometimes unsatisfactory. One promising strategy for treating diseases, particularly cancer, is to target epigenetic factors which are important moderators in physiological and pathogenic networks and they can cooperate with several molecular pathways. The role of lincRNAs as epigenetic factors implicated in the onset and progression of cancer has been underlined by mounting evidence, which has also concentrated on their relationship to miRNAs as their downstream targets (Khorkova et al. 2015; Jiang et al. 2019). The diverse characteristics of the lincRNAs render them highly desirable targets for therapeutic purposes, such as their accessibility to numerous protein targets which were considered undruggable as well as they are easily controlled using synthetic antisense oligonucleotides. In the past, attempts to develop small-molecule medications that modulate cancer-associated proteins have not been very fruitful. Nowadays targeting lincRNAs is achieved via advanced techniques, such as ASOs, CRISPR-Cas9, and nanomedicines. Malignancy is one of the fields where lncRNA-based treatments are currently being explored (Wahlestedt 2013, Kumar and Goyal 2017, Aborehab et al. 2023). Thus, a comprehensive understanding of the regulatory framework of these lincRNAs is crucial to establishing novel treatment approaches. Herein, we briefly discussed the recent findings in the realm of lincRNAs as therapeutic targets in lung diseases as shown in Table 4.

**Table 4:** The lincRNAs as therapeutic targets in lung diseases

LincRNAs	Disease	Regulation	Targeted miRNA	Targeted proteins	Therapeutic intervention	Ref
LINC00511	NSCLC	Up	miR-625-5p	GSPT1	Suppress	(Cheng et al. 2021)
	SCLC	Up	miR-150-5p	TADA1		(Wu et al. 2020a, b)
LINC00115	LAD	Up	miR-154-3p	SP3	Suppress	(Sun et al. 2023)
	Lung cancer		miR-607	ITGB1		(Wu et al. 2022)
	Lung cancer		-	N-cadherin, Vimentin, Fibronectin, Ecadherin		(Shao et al. 2021a)
	LAD		-	IL-6, JAK1, STAT1		(Cai et al. 2022)
MALAT-1	NSCLC	Up	miR-181a-5p	PBOV1	Suppress	(Chen et al. 2022)
			miR-613	COMMD8		(Wang et al. 2020a, 2020b, 2020c)
			miR-515-5p	EEF2		(Rong et al. 2020)
			miR-185-5p	MDM4		(Zhang et al. 2020a, 2020b)
			miR-200a	ZEB1		(Feng et al. 2019)
	Lung cancer		miR-206	MCP-1		(Li et al. 2024)
	Bronchopulmonary dysplasia		-	STING CREB		(Chen et al. 2020)
	PAH		miR-124-3p.1	KLF5		(Wang et al. 2019a, b)
	LAD		miR-140	PD-L1		(Li et al. 2023)
MIAT	Lung cancer	Up	miR-34a	PI3KAkt	Suppress	(Fu et al. 2018)
	NSCLC		-	-		(Pei et al. 2018)
			miR-149-5p	FOXM1		(Zhang et al. 2020a, b)
			miR-184	SF1		(Wu et al. 2020a, b)
			miR-150	ZEB1		(Zhang et al. 2017a, b)
H19	Lung cancer	Up	miR-200a	ZEB1 & 2	Suppress	(Zhao et al. 2019)
	NSCLC		miR-21	TGF-β1		(Zhou et al. 2017)
	Pulmonary fibrosis					(Wang et al. 2019b)
BCYRN1	NSCLC	Up	miR-149	PKM2	Suppress	(Wang et al. 2020a, 2020b, 2020c)
XIST	NSCLC	Up	miR-186-5p		Suppress	(Wang et al. 2017)
			miR- 16-5p	WEE1		(Du et al. 2021)
LincROR	NSCLC	Up	-	AKT, mTOR	Suppress	(Shi et al. 2017)
UCA1	LAD	Up	miR-383	VEGFA	Suppress	(Tang et al. 2023)
	Lung cancer		-	-		(Wang et al. 2015)
	Lung cancer		miR-143	MAPK 1		(Jun et al. 2018)
	Pneumonia		-	EZH2, HOXA1, NF-κB p-65		(Zhang et al. 2022)
PVT-1	NSCLC	Up	-	EZH2, LATS2	Suppress	(Wan et al. 2016a, b)
TUG-1	COPD	Down	miR-145-5p	-	Suppress	(Gu et al. 2019)
MEG3	NSCLC	Down	miR-543	IDO	Restore	(Wang et al. 2021)
			-	MDM2, p53	Restore	(Lu et al. 2013)
			miR-21-5p	PI3K, AKT		(Lv et al. 2021)
			-	DKC1		(Wang et al. 2020a, 2020b, 2020c)
FENDRR	Lung fibrosis	Down	miR-214	TGF-*β*1, SMAD3	Restore	(Huang et al. 2020)
BANCR	NSCLC	Down	-	-	Restore	(Yang and Liu 2019)
LincRNA p21	NSCLC	Down	miR-17-5p	-	Restore	(Ao et al. 2019)

[BANCR: BRAF activated non-coding RNAs; EMT: Epithelial mesenchymal transition; FENDRR: Fetal-lethal non-coding developmental regulatory RNA; FOXM1: Forkhead box M1; GSPT1: G1-S phase transition1; LAD: lung adenocarcinoma; MALAT-1: Metastasis associated lung adenocarcinoma transcript 1; MAPK: Mitogen-activated protein kinase; MEG3: Maternally expressed gene 3; MIAT: Myocardial infarction associated transcript; NSCLC: Non-small cell lung cancer; PAH: pulmonary artery hypertension; PVT-1: Plasmacytoma Variant Translocation 1; SF-1: splicing factor 1; SCLC: small cell lung cancer; TADA1: Transcriptional Adaptor1; WEE: WEE1 G2 Checkpoint Kinase.]

### Oncogenic LincRNAs in lung diseases

**Fig. 3:**
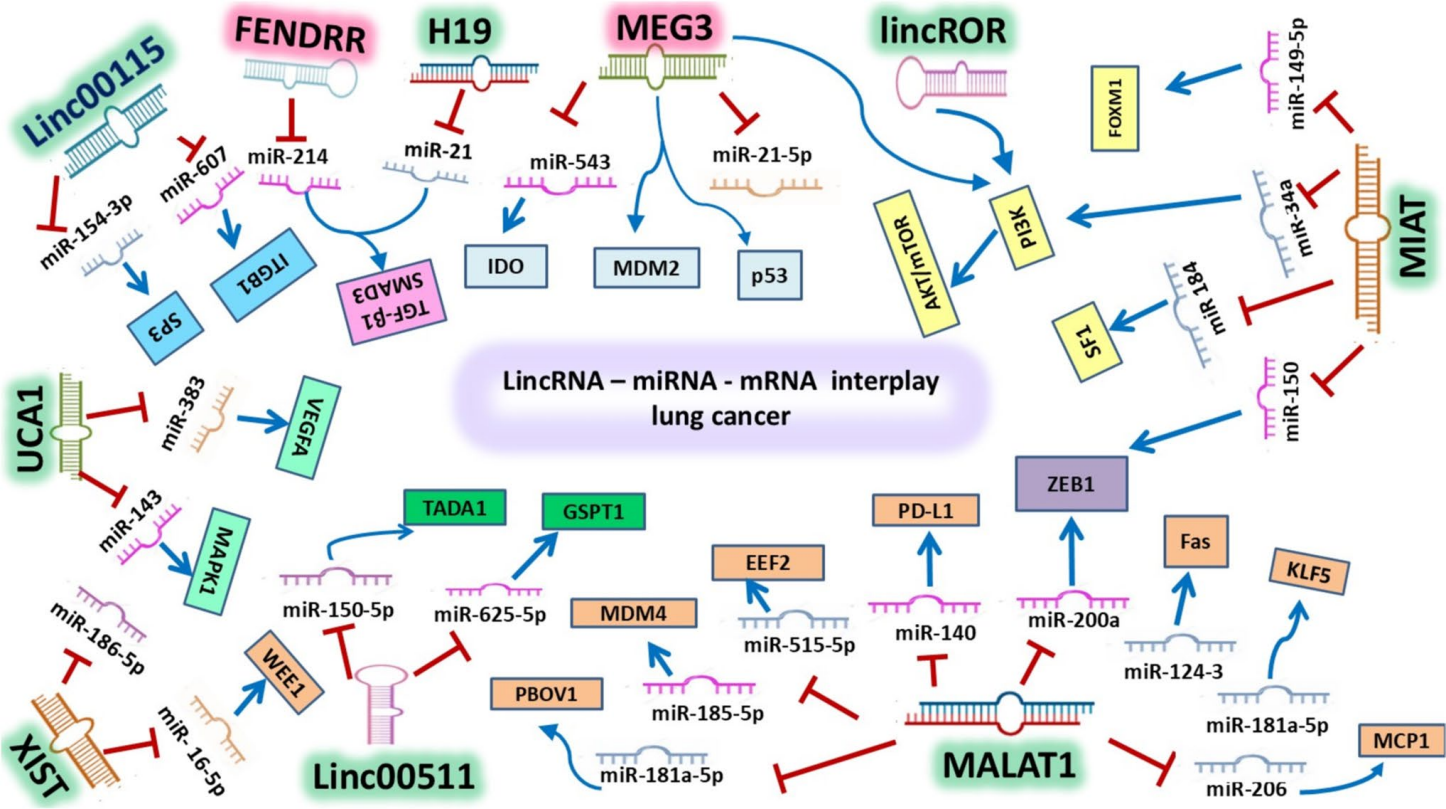
A schematic diagram for oncogenic and tumor suppressor lincRNAs and their downstream miRNA‐mRNA circuit. [LincRNAs: long intergenic noncoding RNAs; miRNA: microRNA; mRNA: messenger RNA.]

Highly expressed oncogenic lincRNAs (Fig. 3) trigger cancer invasiveness, proliferation, angiogenesis, metastasis, and EMT.

**LINC00511** was frequently studied in diverse lung diseases. LINC00511 is upregulated in NSCLC and coupled with aggressive stages and bad prognosis. LINC00511 performs its oncogenic role via stimulating G1-S phase transition1 (GSPT1) and adsorbing the miR-625-5p (Cheng et al. 2021). In LSCC, the LINC00511/ Transcriptional Adaptor1 (TADA1) was highly expressed along with a marked reduction of miR-150-5p (Wu et al. 2020a, b). Another study revealed that LINC00115 acted as a sponger of miR-607 and stimulated ITGB1 resulting in the promotion of lung cancer (Wu et al. 2022). LINC00511 participate in lung cancer mesenchymal transformation and metastasis by modifying EMT-linked genes (Shao et al. 2021a). Cai and his coworkers declared that the highly expressed LINC00115 in LAD participates in cancer growth and spread through modulating JAK1/STAT1 trajectory (Cai et al. 2022).

**MALAT-1,** the oncogenic lincRNA, seems to be a crucial biological modulator that is closely associated with the onset, spread, and responsiveness to the treatment of cancer as well as has been implicated in the interruption of cellular homeostasis. In LADs, it was highly expressed and plays various detrimental functions through different axes, thus MALAT-1 was taken into consideration as a potential therapeutic target. Regarding NSCLC, MALAT-1 fostered the proliferation and invasion of cancerous cells (Schmidt et al. 2011a). MALAT-1 promotes sensitivity to chemotherapeutics via competitive inhibition of miR-181a-5p (Chen et al. 2022). Wang et al. study suggested that suppression of MALAT-1 leads to a further reduction in the transcription of the oncogenic gene COMMD8 via modulating the miR-613 (Wang et al. 2020a, b, c). Rong and his colleagues illustrated that reducing the expression of MALAT-1 could be valuable in fighting NSCLC by attenuating its invasiveness and proliferative properties. MALAT-1 sponges the miR-515-5p and stimulates EEF2, thus proposing its potential as a tailored treatment in NSCLC (Rong et al. 2020). The simultaneous activation of MALAT-1 and MDM4 along with inhibition of the miR-185-5p was observed also in NSCLC (Zhang et al. 2020a, b). Similarly, MALAT-1 was elevated in cell lines of NSCLC and promotes ZEB1 via targeting miR-200a (Feng et al. 2019).

In lung cancer, Li et al. found that MALAT-1 inhibits miR-206 leading to further stimulation of MCP-1 (Li et al. 2024). In bronchopulmonary dysplasia, Chen and his coworkers demonstrated that STING and CREB could be downstream targets of MALAT-1 (Chen et al. 2020). In PAH, MALAT-1 was highly expressed resulting in the inhibition of the miR-124–3 and activation of KLF5 (Wang et al. 2019a, b). In LAD, MALAT-1 blockage exerts an antiproliferation effect via modulating miR-140/PD-L1 (Li et al. 2023).

**MIAT** is an important target in the treatment of different diseases via influencing different cellular signaling (Abdelmonem et al. 2021). MIAT suppression prolonged the survival time and alleviated chemotherapeutic resistance via binding to miR-34a which subsequently modulates PI3K/Akt (Fu et al. 2018). MIAT in NSCLC encourages transcription of Forkhead box M1 (FOXM1) by hindering the miR-149-5p (Zhou, Zhang et al. 2020a, b). MIAT silencing suppresses ZEB1 resulting in attenuation of NSCLC invasiveness. MiRNA-150 plays a potential role in modifying MIAT/ZEB1 activity (Zhang et al. 2017a, b).

**H19** exerts its unfavorable effect on lung cancer cells through stimulating transcription of ZEB1 and 2, the target genes of the miR-200a. In other words, antagonizing miR-200a promotes the performance of ZEB1 and 2, which facilitates the proliferation and invasiveness of the tumor (Zhao et al. 2019). On the other side, the interaction between lincRNA H19 and miR-21 could be a suitable goal in therapeutic strategies of NSCLC as stated by Zhou and his colleagues especially since they were linked with aggressive cancer stages (Zhou et al. 2017). Moreover, this could be based on the universality of miR-21 in various cancer types (El‐Aziz et al. 2023, Eldosoky et al. 2023; Hammad et al. 2023, 2024) and its bioinformatics identified and known downstream genes and/or proteins that are targeted. Additionally, the therapeutic usage of H19 in pulmonary fibrosis was highlighted where silencing of H19 enhanced the expression of the miR-140 which finally repressed TGF-*β*1 (Wang et al. 2019b).

**XIST** lincRNA in NSCLC functional analysis revealed that knocking it down exhibits antiproliferative and anti-invasive effects (Abd-Elmawla et al. 2023). Wang et al. also revealed that these effects are attributed to the inverse association between XIST and miR-186-5p (Wang et al. 2017). Knocking XIST lessens the viability of NSCLC via modulating the miR-16-5p and WEE1 G2 checkpoint kinase (Du et al. 2021).

**BCYRN1** highly expressed was implicated in promoting glycolysis in cancerous tissues via modulating miR-149/PKM2 (Wang et al. 2020a, 2020b,  2020c).

LincROR targeting was reported as an important mechanism and essential goal for controlling NSCLC via modulating the AKT/mTOR axis. Suppressing lincROR showed favorable effects in mitigating NSCLC and promoting sensitivity to drugs (Shi et al. 2017).

**UCA1** suppression lessens malignant cell surveillance by stimulating the activity of the miR-383. Notably, the suppression of miR-383 promotes the development and progression of LAD by targeting the VEGFA gene and reducing apoptosis (Tang et al. 2023). In a clinical investigation on NSCLC patients, UCA1 was markedly associated with the cancer stage, the extent of the metastatic effect, and other clinical data (Wang et al. 2015). Elevated levels of UCA1 are connected with aggressive stages, programmed cell death, and activation of NF-κB p-65 (Zhang et al. 2022).

**PVT-1** is an oncogenic lincRNA that participates in the development of lung cancer by influencing vital processes like angiogenesis, EMT, programmed cell death, and others. Huang and his colleagues documented the high levels of PVT-1 in SCLC as well as its significant associations with pathological stages, metastasis, and prognosis, thus its depletion could exert promising effects in the treatment strategies (Huang et al. 2016). Additionally highly expressed PVT-1 was also associated with aggressive stages and bad prognosis in NSCLC, so it could be a crucial target in the therapeutic interventions of NSCLC (Wan et al. 2016a, b).

**TUG-1** was elevated in COPD, while its depletion mitigates inflammatory reactions via adsorbing the miR-145-5p, thus being valuable in treating such diseases (Gu et al. 2019).

### Tumor suppressor LincRNAs in lung diseases

**MEG3** in NSCLC, was markedly depressed along with high levels of miR-543 and IDO. Restoration of MEG3 could be valuable in the management of NSCLC (Wang et al. 2021). Lu and his colleagues also declared that overexpression of MEG3 inhibits NSCLC via promoting apoptotic reactions. Notably high levels of MEG3 interact with MDM2 and p53 (Lu et al. 2013). Lv et al. stated that the protective effects of MEG3 are achieved by depleting miR-21-5p and PI3K/AKT along with promoting PTEN level (Lv et al. 2021). Another study reported that high levels of MEG3 could exert its therapeutic effect in ameliorating NSCLC via suppressing DKC1 (Wang et al. 2020a, 2020b, 2020c).

**FENDRR** lincRNA was also established as an upstream regulator of TGF-*β*1/SMAD3 in lung fibrosis. Furthermore, FENDRR was rationalized as an anti-fibrotic molecule where it suppresses the profibrotic miR-214 (Huang et al. 2020).

**BANCR** restoration suppressed the invasiveness and surveillance of NSCLC cells (Yang and Liu 2019) (Fig. 3).

**LincRNA p21** is known as one of the tumor-suppressing genes in NSCLC. Stimulating the expression of lincRNA p21 could be considered a framework in the treatment of NSCLC (Castellano et al. 2016). LincRNA p21 elevation exerts antiproliferative and antimigration effects, while its knockdown showed the opposite. An inverse link was detected between the expression of lincRNA p21 and the oncogenic miR-17-5p (Ao et al. 2019).

## Limitations

Not all known lincRNAs were studied for their association with all cancer types (including lung cancer as a once crucial type of lung disease. Moreover, the potential of lincRNAs as lung cancer diagnostic markers should be further clarified. Combining ncRNA-based diagnostics or treatments with chemotherapy, immunotherapy, or existing biologics for asthma or COPD should be further investigated. Furthermore, comprehensive data regarding the mechanisms by which lincRNAs contribute to the success of therapeutic interventions or the improvement of patient outcomes should be additionally elucidated.

## Future perspective for sustainability

RNAi-knockdown of various lincRNAs to observe more the tumor immune microenvironment (TIME) via various immune cells reprograming (Elanany et al. 2023) as well as the lung cancer hallmarks. Moreover, tumor migration, invasion, and metastasis tracing and identification in various types of lung cancer SCLC or NSCLC cells in relation to lincRNAs. Third, identify the expression of potential downstream genes involved in cell adhesion and/or metastasis or angiogenesis (Chiang et al. 2024). As well as exploring if supplementing tumor-modulating vitamins as D (Chen et al. 2023) could influence lincRNAs-related TME.

## Recommendation

Designing more lincRNA-specific anti-sense oligonucleotides for in vivo lung diseases, especially lung cancer model treatment.

Second, the need for experimental validation of computer-research findings as potential future recommended implementation of these findings from the lab to the hospital bed side.

## In summary

**Fig. 4:**
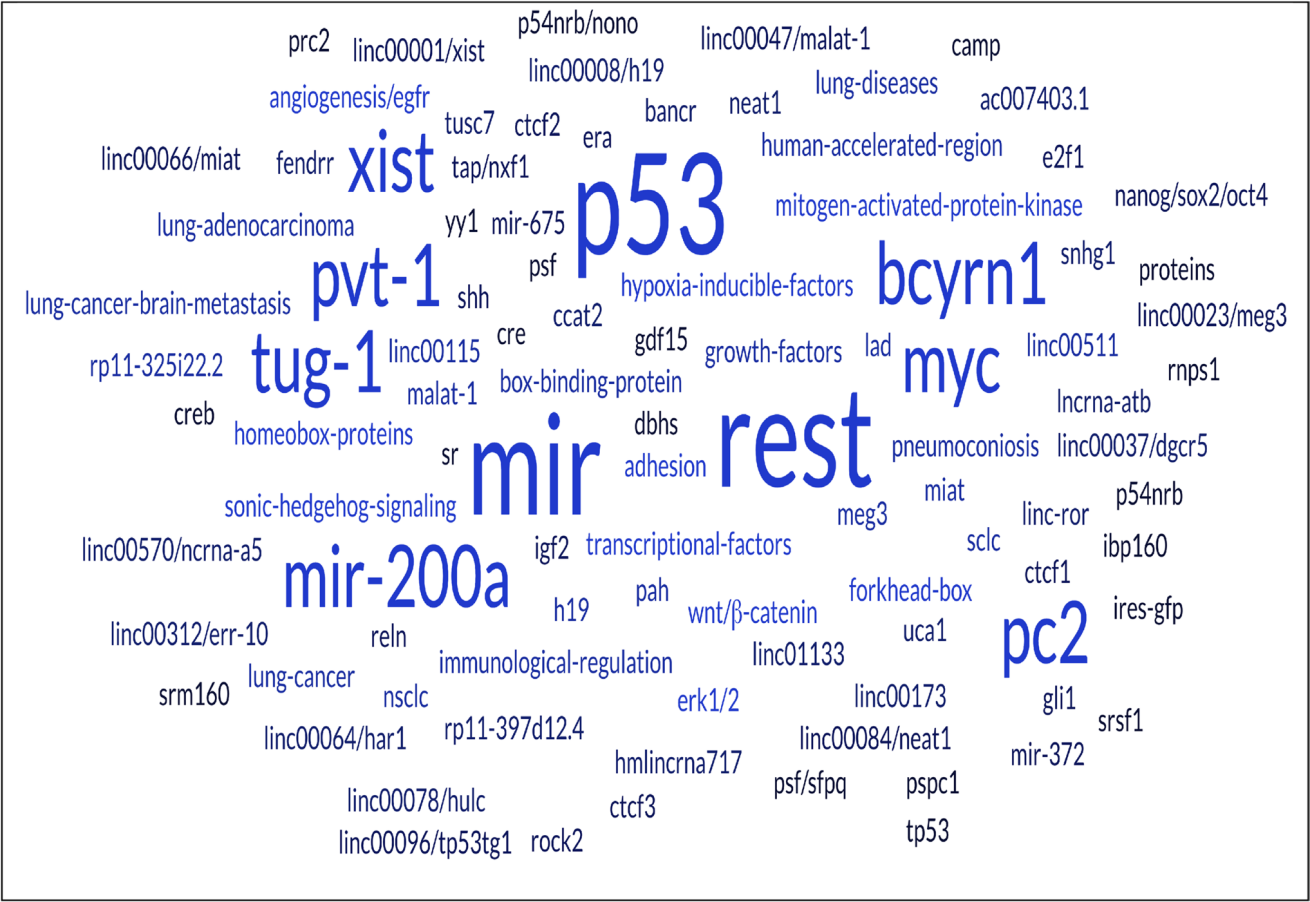
Wordcloud presenting (135 words) of various lincRNAs in all lung diseases reviewed with their downstream miRs and protein/genes as well as the corresponding molecular pathways/mechanisms. https://www.freewordcloudgenerator.com/generatewordcloud Accessed Jan. 4.^th^ 2025

Unraveling lincRNA’s roles in lung diseases and lung cancer pathogenesis/risk via highlighting their downstream miRs/protein or gene axes molecular pathways/ mechanisms influence the lung disease processes (Fig. 4 and Supplementary Fig. S1 for the most common lincRNAs). The current review raises a flag to highlight the necessity for the development of lincRNA-related diagnostic(s)/prognostic(s) precision as well as next-generation therapies.

## Conclusion

LncRNAs, particularly lincRNAs, exhibit significant roles in the pathogenesis of various lung diseases and lung cancer. A comprehensive investigation of the specific lincRNAs and their interactions with other miRNAs, genes, and proteins may facilitate the identification of novel strategies for the development of personalized therapeutic interventions and the improvement of patient outcomes.

## Supplementary Information

Below is the link to the electronic supplementary material.

Supplementary file1 (DOCX 1070 KB)

## Data Availability

No datasets were generated or analysed during the current study.

## Abbreviations

ANRIL: Antisense Noncoding RNA in the INK4 Locus
ASO: Antisense oligonucleotides
BANCR: BRAF activated non-coding RNAs
Bcl-2: B-cell lymphoma 2
BCYRN1: Brain Cytoplasmic RNA 1
CCAT2: Colon cancer-associated transcript 2
CEA: Carcinoembryonic Antigen
*CELF1*: CUGBP and Elav-like family member 1
ceRNA: Competing endogenous RNA
COPD: Chronic Obstructive Pulmonary Disease
CRE: CAMP response element
CREB: Cyclic AMP-responsive element binding
CTR1: Copper transporter 1
CYTOR: Cytoskeleton regulator RNA
DBHS: Drosophila melanogaster behavior, human splicing family
DFS: Disease-Free Survival
DGCR5: DiGeorge syndrome-associated ncRNA
EGFR: Epidermal growth factor receptor
EMT: Epithelial-mesenchymal transition
*ERBB4*: Erb-b2 receptor tyrosine kinase 4
ERR-10: Estrogen-related receptor
Etk: Epithelial Tyrosine Kinase
EZH2: Enhancer of zeste homolog 2
FENDRR: Fetal-lethal non-coding developmental regulatory RNA
FGF2: Fibroblast growth factor 2
FOXF1-AS1: Forkhead Box F1 Antisense 1
FOXM1: Forkhead box M1
FSCN1: Fascin Actin-Bundling Protein 1
GDF15: Growth Differentiation Factor 15
GSPT1: G1-S phase transition1
HIF1A-AS1: Hypoxia inducible factor 1 subunit alpha-antisense 1
HOXB7: Homeobox B7
HULC: Hepatocellular Carcinoma Up-Regulated Long Non-Coding RNA
KLF2: Krüppel-like Factor 2
LAD: Lung adenocarcinoma
LATS2: Large tumour suppressor kinase 2
LINC-PINT: LincRNA-P53-Induced Transcript
LINC-ROR: LincRNA-Regulator Of Reprogramming
LincRNA: Long Intergenic Non-Coding RNA
LncRNA: Long non-coding RNAs
lncRNA-ATB: LncRNA-activated by TGF-β
LSCC: Lung squamous cell carcinoma
MALAT-1: Metastasis associated lung adenocarcinoma transcript 1
MAPK9: Mitogen-activated protein kinase 9
MEG3: Maternally expressed gene 3
MIAT: Myocardial infarction-associated transcript
miR: MicroRNA
ncRNA: Non-Coding RNA
NEAT1: Nuclear enriched abundant transcript 1
NSCLC: Non-small cell lung cancer
OS: Overall Survival
PAH: Pulmonary arterial hypertension
Pc2: Polycomb 2 protein
PRC2: Polycomb repressive complex 2
PVT-1: Plasmacytoma Variant Translocation 1
REST: Repressor element 1-silencing transcription factor
SCLC: Small Cell Lung Cancer
SF-1: Splicing factor 1
SHH: Sonic Hedgehog
siRNA: Small interfering RNA
Skp2: S-phase kinase-associated protein 2
SNHG1: Small nucleolar RNA host gene 1
SNPs: Single nucleotide polymorphisms
SPRY4-IT1: Sprouty RTK signaling antagonist 4-intronic transcript 1
SRSF1: SR-rich splicing factor 1
TADA1: Transcriptional Adaptor1
TDP-43: Transactive response DNA binding protein of 43 kDa
TKIs: Tyrosine kinase inhibitors
*TP53*: P53 gene
*TP53TG1*: TP53 target gene 1
TUG-1: Taurine-upregulated gene 1
TUSC7: Tumor suppressor candidate 7
UCA1: Urothelial cancer associated 1
WEE1: WEE1 G2 Checkpoint Kinase
XIST: X inactive specific transcript
XWLC: Xuanwei lung cancer
