# Supplementary material for: Comprehensive insights and In silico analysis into the emerging role of LincRNAs in lung diseases pathogenesis; a step toward ncRNA precision
Source: Funct Integr Genomics. 2025 Feb 6;25(1):34. doi: 10.1007/s10142-025-01540-1 (PMC11802690; doi:10.1007/s10142-025-01540-1)
Supplement: Supplementary file 1 — Supplementary file1 (DOCX 1070 KB) [file 10142_2025_1540_MOESM1_ESM.docx]

| A | B | C | |
| --- | --- | --- | --- |
| 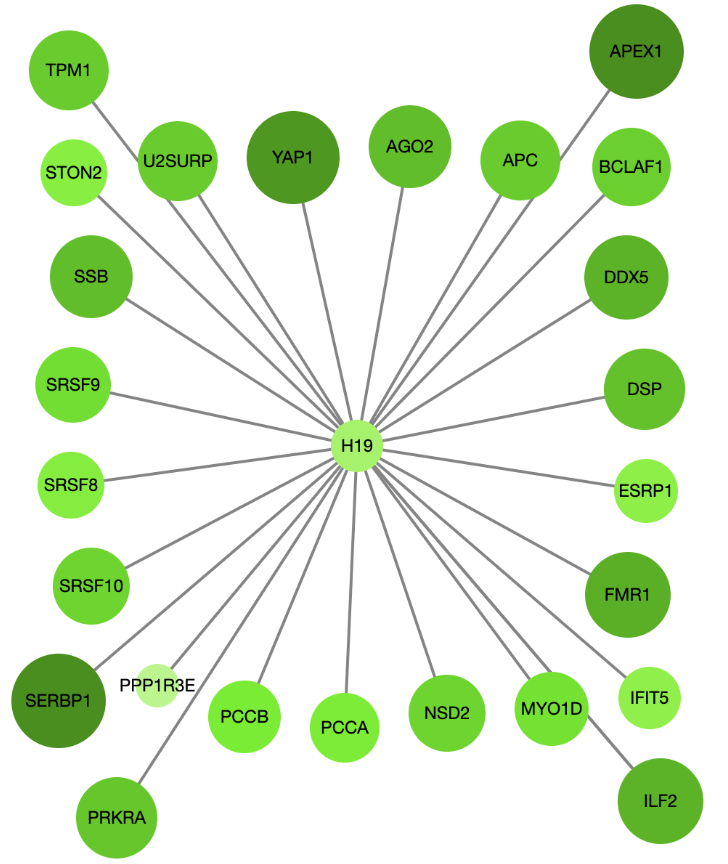 | 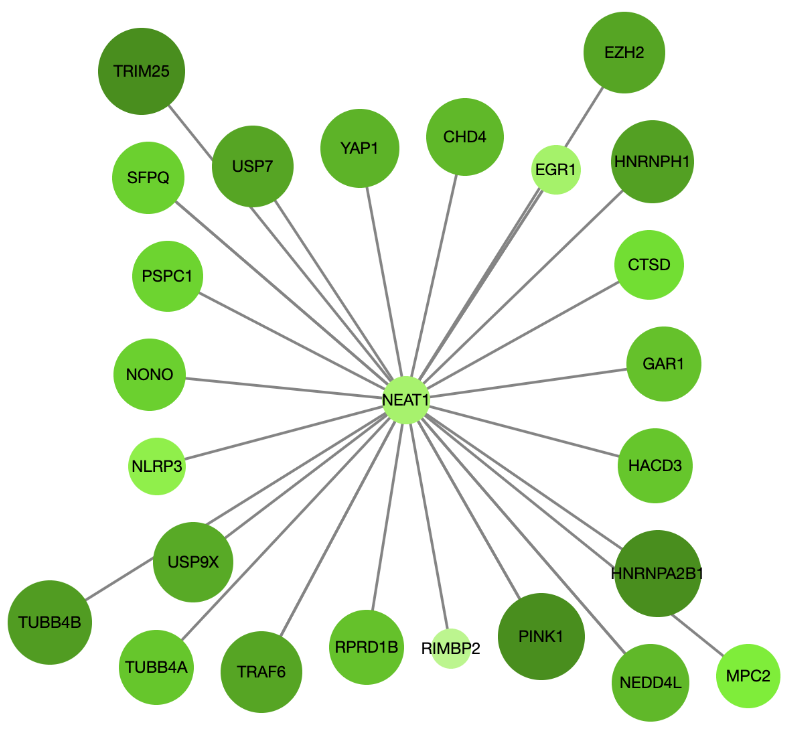 | | 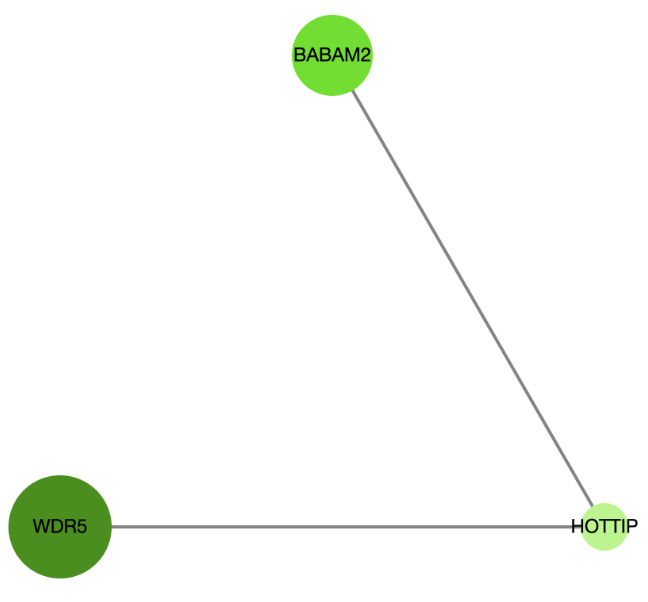 |
| <https://ctdbase.org/tools/cyjs.go?type=gene&report=gg&window=yes&color=91&terms=283120> and  <https://thebiogrid.org/129467/summary/homo-sapiens/h19.html> | <https://ctdbase.org/tools/cyjs.go?type=gene&report=gg&window=yes&color=91&terms=283131> and <https://thebiogrid.org/129471/summary/homo-sapiens/neat1.html> | <https://ctdbase.org/tools/cyjs.go?type=gene&report=gg&window=yes&color=91&terms=100316868> and <https://thebiogrid.org/1147673/summary/homo-sapiens/hottip.html> | |
| D | |  | |
| 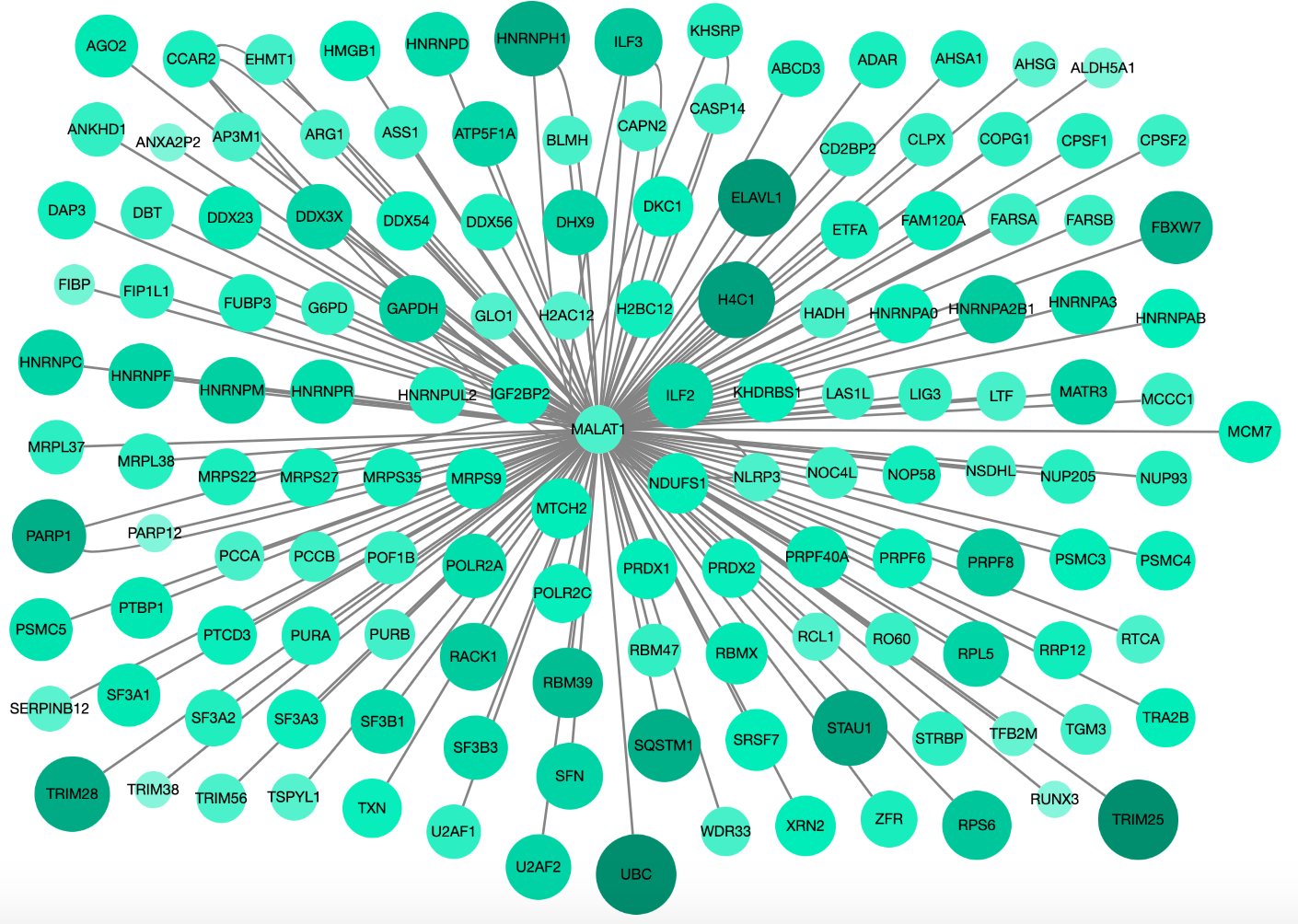 | |  | |
| <https://ctdbase.org/tools/cyjs.go?type=gene&report=gg&window=yes&color=91&terms=378938> and <https://thebiogrid.org/132077/summary/homo-sapiens/malat1.html> | |  | |

**Supplementary Fig. S1. The most common lincRNAs in lung cancer** gene-to-gene interaction via ctd; [**Comparative Toxicogenomics Database**](https://ctdbase.org/) <https://ctdbase.org/> and the BioGRID^4.4^ <https://thebiogrid.org/> Accessed Jan. 4^th^ 2025.
